# Exposing the cellular situation: findings from single cell RNA sequencing in breast cancer

**DOI:** 10.3389/fimmu.2025.1539074

**Published:** 2025-03-06

**Authors:** Gaofeng Ni, Xinhan Li, Wenyang Nie, Zhenzhen Zhao, Hua Li, Hongyan Zang

**Affiliations:** ^1^ Department of Breast Surgery, Yantaishan Hospital Affiliated to Binzhou Medical University, Yantai, China; ^2^ The First Clinical Medical College, Shandong University of Traditional Chinese Medicine, Jinan, Shandong, China; ^3^ Department of General Surgery, Affiliated Hospital of Youjiang Medical University for Nationalities, Baise, Guangxi, China; ^4^ Key Laboratory of Tumor Molecular Pathology of Baise, Affiliated Hospital of Youjiang Medical University for Nationalities, Baise, Guangxi, China

**Keywords:** single cell RNA sequencing, breast cancer, CEBPD, transcription factors, tumor microenvironment, metabolism

## Abstract

**Background:**

Breast Cancer (BC) ranks among the top three most prevalent cancers globally and stands as the principal contributor to cancer-related fatalities among women. In spite of the substantial occurrence rate of BC, the early stage of this disease is generally regarded as curable. However, intra-tumor heterogeneity presents a formidable obstacle to the success of effective treatment.

**Method:**

In this research, single cell RNA sequencing was utilized to dissect the tumor microenvironment within BC. Slingshot, CytoTRACE and Monocle 2 were applied to illustrate the differentiation process of each subpopulation in the pseudotime sequence. To comprehensively comprehend the tumor cells (TCs) in BC, an analysis of upstream transcription factors was carried out via pySCENIC, while downstream pathway enrichment was conducted through KEGG, GO and GSEA. The prognosis model was established based on the bulk data obtained from TCGA and GEO databases. Knock-down experiments were also implemented to explore the function of the transcription factor *CEBPD* in the TCs.

**Results:**

Our in-depth analysis identified eight principal cell types. Notably, TCs were predominantly found within epithelial cells. The classification of TCs further uncovered five unique subpopulations, with one subpopulation characterized by high *UGDH* expression. This subpopulation was shown to possess distinct metabolic features in metabolism-related investigations. The intricate communication modalities among different cell types were effectively demonstrated by means of CellChat. Additionally, a crucial transcription factor, *CEBPD*, was identified, which demonstrated a pronounced propensity towards tumors and harbored potential tumor-advancing characteristics. Its role in promoting cancer was subsequently verified through *in vitro* knock-down experiments. Moreover, a prognostic model was also developed, and a risk score was established based on the genes incorporated in the model. Through comparing the prognoses of different UTRS levels, it was determined that the group with a high UTRS had a less favorable prognosis.

**Conclusion:**

These outcomes contributed to the elucidation of the complex interrelationships within the BC tumor microenvironment. By specifically targeting certain subpopulations of TCs, novel treatment strategies could potentially be devised. This study shed light on the direction that future research in BC should take, furnishing valuable information that can be utilized to enhance treatment regimens.

## Introduction

Breast cancer (BC) is among the top three most frequently occurring cancers globally and represents the leading cause of cancer mortality among women (1, 2). According to the latest data from the World Health Organization, in 2022, 2.3 million women worldwide will be diagnosed with breast cancer and 670,000 will die from it. Global estimates show alarming inequalities in the burden of breast cancer in terms of human development. For example, in countries with a high human development index, 1 in 12 women will be diagnosed with breast cancer in their lifetime, and 1 in 71 women will die from breast cancer. In the context of all malignant tumors in China, the incidence of BC occupies the fifth position overall but holds the top spot among women. Despite the relatively high incidence of early-stage BC, it is generally regarded as being curable. The principal obstacle to the success of effective treatment lies in intratumor heterogeneity (3, 4). BC can be classified into at least four clinically significant molecular subpopulations, namely ductal A, ductal B, HER2-enriched, and basal-like. Through gene copy number and expression analysis, more than a dozen distinct molecular subpopulations have been identified (5). Among these subpopulations, ER+/HER2- BCs make up approximately 60% to 70% of all BCs (6).

The exact etiology of BC is still not fully understood. However, early-stage BC that is restricted to the axillary lymph nodes is considered to be curable. In contrast, advanced metastatic BC is currently incurable, and the focus of treatment is mainly on prolonging survival and managing symptoms rather than enhancing the quality of life (7). BC patients typically have a poor prognosis due to highly aggressive tumors and the absence of early warning signs, which makes treatment extremely challenging. Despite recent advancements in BC treatment, only a small number of patients can reap the benefits of the available options because of the high recurrence rate and the limitations of post-metastatic drugs and therapies. Consequently, it is essential to explore new and innovative therapeutic strategies for BC.

Moreover, the processes responsible for tumor growth, metastasis, treatment resistance, and immunosuppression are concealed by the intricacy and diversity of malignancies. To achieve effective tumor control and management, it is necessary to have an in-depth comprehension of the molecular pathways that underlie carcinogenesis and tumor development. Both the cancer epithelial intracellular factor (8–10) have an impact on the progression of BC. The epithelial-mesenchymal transition (EMT) converts epithelial cells into mesenchymal cells, which promotes the formation of tumor stem cells and augments the infiltration, migration, and metastasis of tumor cells (TCs). Previous investigations have already shown that the number of cells expressing EMT-characteristic genes is substantially increased in triple-negative breast cancer (TNBC) and ER+ BCs (11). Investigating epithelial cells holds great significance for devising strategies to restrain BC invasion and metastasis, reducing the mortality rate among BC patients, and uncovering novel treatment targets. Directing therapeutic endeavors towards the TME could prove to be of utmost importance for future cancer treatments, given that the diverse cell types within the TME have considerably spurred oncology research. The TME has played a vital role in the development and progression of BC (12, 13). The TME represents a complex network that surrounds BC cells and consists of numerous cellular components such as fibroblasts, endothelial cells, and immune cells, as well as extracellular elements like cytokines, hormones, extracellular matrix, and growth factors (14). These various components have an impact on the biological behavior of cancer and the efficacy of treatment (1, 15, 16).

Within the TME, epithelioid cells are a type of stem cells that undergo EMT. During this process, they shed their original morphological and functional characteristics and transform into mesenchymal-like cells (17). EMT serves as a crucial step in tumor development. TCs that experience EMT acquire invasive capabilities, allowing them to penetrate the surrounding stroma and establish a microenvironment that is favorable for tumor growth and metastasis. Metabolically, EMT-induced TCs show significant alterations. There is a notable shift in energy metabolism, with an increased preference for glycolysis over oxidative phosphorylation. This metabolic reprogramming provides the cells with a more rapid supply of ATP, which is essential for their enhanced migratory and invasive activities.

EMT is associated with tumor invasion, metastasis, and the emergence of stem cell-like properties. Previous studies have pointed out that EMT is a characteristic hallmark of BC (18).

Recent advancements in high-throughput scRNA-seq have demonstrated its ability to analyze heterogeneous tumors, elucidate the transcriptomic characteristics of cancer cells and their microenvironment, and explicate the interactions between cancer cells and the components of the microenvironment (19–21). This forms the basis for broadening our understanding of cancer and formulating effective early detection and treatment strategies. Although single cell transcriptome analysis has been previously employed to investigate the BC TME, its application in the context of BC is currently limited.

In this study, scRNA-seq was employed to analyze BC samples. The objective was to clarify the immune microenvironment and mapping of BC, thus providing novel perspectives for its treatment. This paper conducts an in-depth exploration of the role played by the subpopulations of TCs in BC as well as their connection with tumor tissue. It offers precious insights into the causes and progression of BC, which is of great assistance in improving treatment approaches.

## Methods

### Data source

The scRNA-seq data pertaining to BC was obtained from the Gene Expression Omnibus (GEO) website (https://www.ncbi.nlm.nih.gov/geo/) under accession number GSE161529. The details regarding patient clinical samples can be accessed at https://www.ncbi.nlm.nih.gov/geo/query/acc.cgi. Given that this study made use of data from a publicly available database, ethical approval was not necessary.

### Single cell RNA sequencing

Upon importing the gene expression data into R, the Seurat software was employed to analyze it (22–26). The quality criteria utilized for cell elimination were as follows: features ranging from 300 to 7,500, count values between 500 and 100,000, mitochondrial gene expression constituting less than 25% of the total gene count, and erythrocyte gene expression accounting for less than 5% of the total gene count.

The “NormalizeData” and “ScaleData” functions in the Seurat R package (27–29) were applied to normalize and standardize the gene expression data. To identify the top 2,000 most variable genes for principal component analysis (PCA), the “FindVariableFeatures” tool was utilized (30–32). The “FindClusters” function was employed to cluster the cells (33, 34), leveraging the first 30 principal components (PCs). PCA was performed using “RunPCA.” Uniform moving approximation and projection (UMAP) was used to reduce dimensionality and visualize gene expression in the top 30 PCs (24, 25). Batch effects in the samples were mitigated using the Harmony R package.

### Identification of cell subpopulations

Cell clusters were identified using the “FindClusters” and “FindNeighbors” functions (35–37) of Seurat (38–40). To evaluate differentially expressed genes (DEGs) in different cell clusters, the “FindAllMarkers” function of Seurat (40–43) was used. The Wilcoxon rank sum test was used in this study.

### Trajectory analysis of TCs subpopulations

Initially, the CytoTRACE tool was used to assess each TCs subgroup’s cellular stemness (38, 39, 44). It is able to reconstruct the developmental trajectory of cells based on single-cell transcriptome data, which is critical for understanding the differentiation pathways of cells during development.

The determination of Cellular Lineages and pseudotimes was carried out through the application of the Slingshot R program. In this process, the branching histories of genealogical structures were modeled by making use of synchronized master curves, and they were further represented with the employment of clustering-based minimal spanning trees.

Subsequently, we initiated the reconstruction of cell differentiation trajectories with the assistance of the Monocle software toolkit (45). These reconstructed trajectories were then downscaled by means of DDRTree, and the progression of the growth of subpopulation cells along the freshly constructed trajectories was monitored. The acquisition of Gene trajectory curves was achieved by utilizing the “getCurves” function. A generalized additive model featuring a negative binomial distribution for each individual gene was employed to scrutinize the correlation between pseudotime and gene expression. This particular technique rendered it feasible to model genes that exhibited slow fluctuations in expression across the entirety of the pseudotime continuum (46).

### Enrichment analysis

Enrichment of DEGs was performed by using Gene Ontology (GO) (47, 48), Kyoto Encyclopedia of Genes and Genomes (KEGG) (49–52), and Genome Sequence Enrichment Analysis (GSEA) tools. These tools can be accessed at the website http://software.broadinstitute.org/gsea/msigdb and used together with the Cluster Profiler R package.

### Cell communication analysis

The CellChat R package (53) was employed to analyze intricate cell-cell interactions and construct regulatory networks based on ligand-receptor expression. By analyzing the ligand-receptor interactions, we were able to infer communication between cells at both the ligand-receptor and signal pathway levels. This is important for understanding the functional consequences of intercellular communication. The “netVisual DiffInteraction” function was used to precisely identify differences in cell communication intensity, while the “IdentifyCommunicationPatterns” function was applied to measure the number of communication patterns. A significance level of 0.05 was set as the cutoff for determining statistical significance. This approach enabled a detailed exploration and quantification of the complex communication dynamics occurring between cells, providing valuable insights into the underlying biological processes and potential regulatory mechanisms.

### pySCENIC analysis

To assess the transcriptional activity within distinct subpopulations of TCs, we resorted to Python’s SCENIC analysis. SCENIC is a tool for reconstructing gene regulatory networks using scRNA-seq data while identifying stable cell states (54). This particular analytical method enabled us to gain a deeper understanding of the regulatory mechanisms governing gene expression in these specific TCs subpopulations. By leveraging SCENIC, we could identify key transcriptional regulators and their associated gene networks, thereby shedding light on the molecular processes that might be driving the behavior and characteristics of different TCs subsets.

### Cell culture

The Cell lines BT-549 and MDA-MB-436 were obtained from the Typical Culture Collection in the United States. For the cultivation of the BT-549 cell line, RPMI1640 medium was used, which was supplemented with 10% fetal bovine serum and 1% penicillin-streptomycin. The culturing environment was maintained at 37°C, with a gas mixture of 5% CO_2_ and 95% humidity. In the case of the MDA-MB-436 cell line, it was cultured in RPMI1640 medium supplemented with 10% FBS, 1% penicillin-streptomycin, and an additional 1% sodium pyruvate. The standard culturing conditions for this cell line were also set at 37°C, with 5% CO_2_ and 95% humidity. These specific culturing protocols were essential for the proper growth and maintenance of the respective cell lines, ensuring the reproducibility and reliability of any experiments or studies conducted using them.

### Cell transfection

The knockdown of *CEBPD* was accomplished by means of small interfering RNA (siRNA) constructs procured from GenePharma (Suzhou, China). The transfection process was carried out in strict accordance with the protocol provided by the manufacturer of Lipofectamine 3000 RNAiMAX (Invitrogen, USA). Cells that had reached 50% confluency in 6-well plates were subjected to transfection using two distinct knockdown constructs, namely Si-*CEBPD*-1 and Si-*CEBPD*-2, along with a negative control (si-NC). The transfection reagent Lipofectamine 3000 RNAiMAX (Invitrogen, USA) was employed in each instance of transfection. This meticulous approach was crucial to ensure the effective and accurate knockdown of *CEBPD*, allowing for the subsequent evaluation of its impact on relevant cellular processes and phenotypes.

### Cell viability assay

The cell viability of the transfected BT-549 and MDA-MB-436 cells was evaluated through the utilization of the CCK-8 assay. After a 24-hour culture period, the cells were seeded at a density of 5 × 10³ per well in 96-well plates. Subsequently, 10μL of CCK-8 reagent (A311-01, Vazyme) was added to each well, and the plates were then incubated at 37°C in the dark for 2 hours. The absorbance at 450 nm was measured on each day from day 1 to day 4 post-transfection using an enzyme marker (A33978, Thermo). The average OD values were then plotted, providing a visual representation of the cell viability over time. This experimental setup and procedure allowed for a quantitative assessment of the impact of the transfection on the viability of the cells, which is crucial for understanding the potential effects of the *CEBPD* knockdown on cell growth and survival.

### 5-Ethynyl-2’-deoxyuridine proliferation assay

In a 6-well plate, 5×10³ transfected BT-549 and MDA-MB-436 cells were seeded into each well and then cultured overnight. A 2x EdU working solution was formulated by mixing serum-free medium with 10 mM EdU. The cells were incubated for 2 hours at 37°C. After that, they were rinsed with PBS and fixed with 4% paraformaldehyde for 30 minutes. Next, a permeabilization step was carried out using a solution containing 2 mg/mL glycine and 0.5% Triton X-100 for 15 minutes. Subsequently, the cells were stained with 1X Apollo and 1X Hoechst 33342 for 30 minutes at room temperature. Finally, fluorescence microscopy was employed to evaluate cell proliferation. This series of steps enabled the visualization and quantification of the proliferative activity of the transfected cells, providing valuable insights into the role of *CEBPD* knockdown in modulating cell growth.

### Wound-healing assay

The stably transfected cells were cultured until they reached confluence within a 6-well plate. Subsequently, each well was scraped carefully with a sterile 200μL pipette tip. To remove any remaining cellular debris, the wells were then rinsed with PBS. After that, the cells were incubated in a serum-free medium. Photographs of the scratches were taken at 0 and 48 hours, and then the widths of the scratches were precisely measured using the Image-J software. This procedure allowed for an assessment of the migratory ability of the stably transfected cells, as changes in the scratch widths over time can indicate how effectively the cells are able to move and fill in the gaps created by the scraping.

### Transwell assay

First, the cells were incubated in serum-free medium for a period of 24 hours. After the Matrigel treatment was carried out, the cell suspension was carefully placed in the upper chamber of the Costar plate. Meanwhile, the lower chamber was filled with serum-enriched medium. Subsequently, the cells were incubated for 48 hours within a culture incubator. To evaluate the invasive ability of the cells, once the incubation was completed, they were fixed using 4% paraformaldehyde. After that, the cells were stained with crystal violet. This series of steps and treatments enabled a determination of how effectively the cells could penetrate through the Matrigel and migrate towards the serum-enriched medium in the lower chamber, thereby providing an assessment of their invasive capabilities.

### Kaplan-Meier survival curves for selected genes

Survival analysis was carried out by us with the utilization of the R software packages known as survminer and survival. These packages offer a range of functions and tools that are highly valuable in analyzing and interpreting survival data.

### Construction and validation of prognostic model

We integrated 10 machine learning algorithms and 101 algorithm combinations. Based on 10x cross-validation, using ten machine learning algorithms: stepwise Cox, random survival forest [RSF], elastic network [Enet], supervised principal components [SuperPC], partial least squares regression for Cox [plsRcox], CoxBoost, survival support vector machine [survival-SVM], Lasso, Ridge, and generalized boosted regression modeling [GBM]) built 101 model combinations. The consistency index (C-index) of the model combinations used (101 in total) across all datasets (including the training set) is calculated and ranked according to the average C-index. Finally, the evaluation results of the models are visualized through heat maps and the most robust and valuable prognostic models are selected.


Risk score=Xi × Yi

In this context, the X denoted the coefficient, while Y represented the level of gene expression. According to the calculated risk score, patients were divided into high risk group and low risk group. The common grouping method was to divide patients according to the optimum cutoff value. Patients with a risk score higher than the optimum cutoff value were classified as high risk group, and those with a risk score lower than the optimum cutoff value were classified as low risk group.

The constructed 101 algorithm combinations was evaluated using a variety of methods. Kaplan-Meier survival analysis was used to compare the survival difference between the high-risk group and the low-risk group, draw the survival curve and calculate the log-rank P value, and evaluate the predictive ability of the model for patient survival. Receiver operating characteristic curve (ROC curve) and area under curve (AUC) were used to evaluate the prediction accuracy of the model (55–57). The closer the AUC is to 1, the better the prediction performance of the model.

### Estimation of immune cell infiltration

The computational analytic tools, namely CIBERSORT (http://cibersort.stanford.edu/), ESTIMATE, and Xcell, were employed to estimate the immune cell infiltration in each BC sample within the TCGA dataset. Subsequently, by means of the CIBERSORT method, a more in-depth examination was conducted regarding the high or low quantity of immune cells in different groups, as well as their associations with OS, modeling genes, and UTRS.

### Somatic mutation analysis

The TCGA database furnished the requisite mutation data for somatic mutation analysis. We examined the distribution of mutations in both the modeled genes and the highly mutated genes. The “maftools” software program was employed to calculate the tumor mutation load (TMB) of each TCs sample. Based on the median TMB value, the TCs samples were divided into high and low TMB groups. Additionally, the Kaplan-Meier method was used to analyze the survival outcomes of the different groups. Moreover, we investigated the copy number variation (CNV) patterns of the modeled genes.

## Results

### Visualization of major cell types and cell subpopulations during BC progression

**Figure 1:**
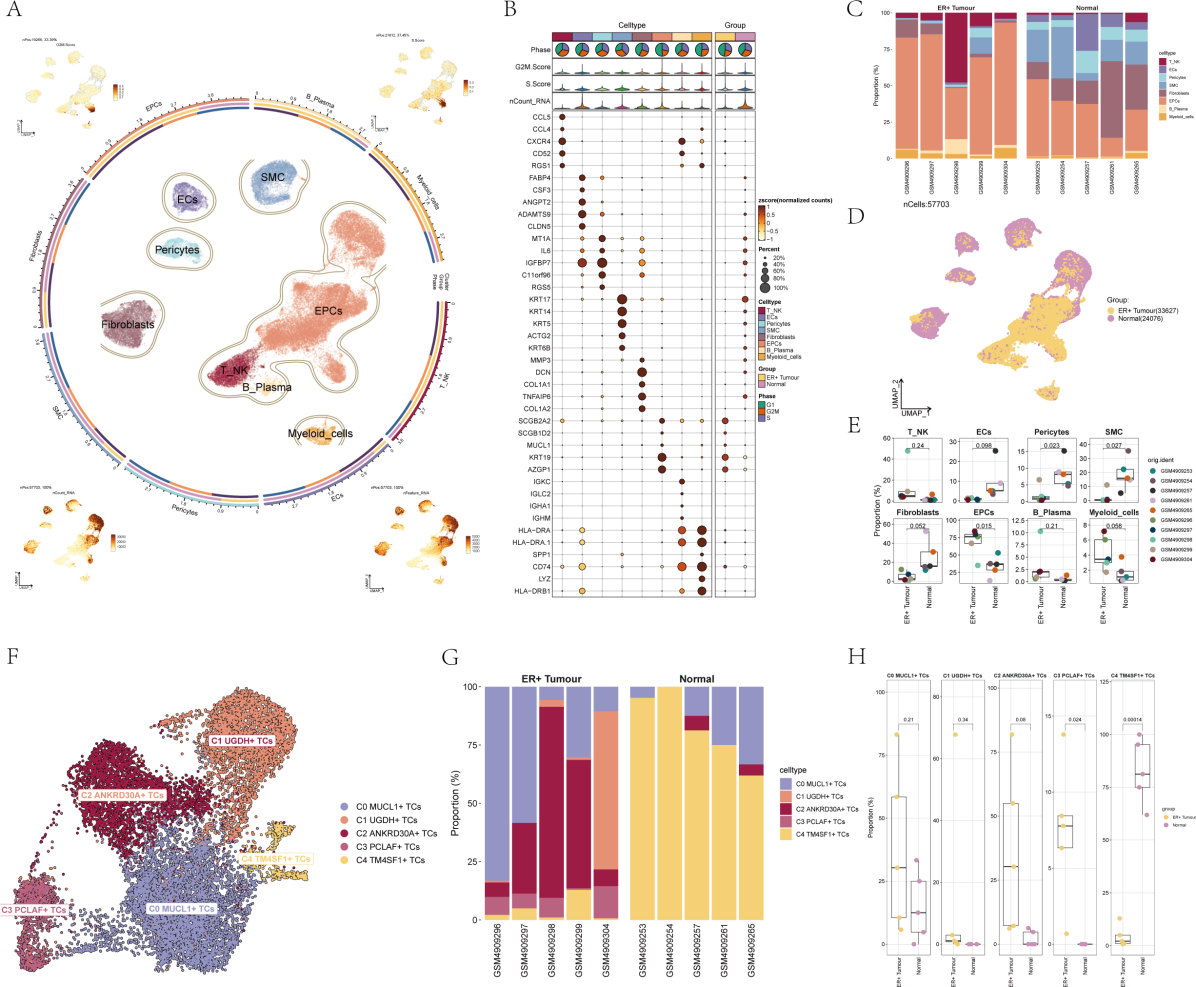
scRNA-seq revealed major cell types during BC progression. **(A)** UMAP plot showed the distribution of 8 cell types of BC patient cells, each point corresponded to a single cell colored according to the cell type. The UMAP plot visualized several relevant features of the 8 cell types of BC patient cells: G2M.score, S.score, nCount_RNA, nFeature_RNA. **(B)** Dot plot showed differential expression of Top5 maker genes in 8 different cell types of BC. The size of the dots indicated the percentage of the gene expressed in the subpopulation, and the shade of the color indicated the expression level of the gene. **(C)** The bar graph showed the expression of cell types in different samples in the ER+ Tumor group and Normal group of BC. Different colors indicated different cell types. **(D)** UMAP plot demonstrated the distribution of cells in ER+ Tumor group and Normal group of BC patients. Each point corresponded to a single cell colored according to group. **(E)** Box line plot demonstrated the expression of different samples between each cell type. **(F)** UMAP plot demonstrated the distribution of five cell subpopulations of BC patient cells, each point corresponded to a single cell colored according to the cell subpopulation. Different colors indicated different cell subpopulations. **(G)** Bar graph demonstrated the expression of cell subpopulations in different samples in ER+ Tumor group and Normal group in BC. **(H)** Box line graph demonstrated the distribution of ER+ Tumor group and Normal group in 5 cell subpopulations.

After meticulous quality control procedures and the elimination of batch effects, a total of 57,703 cells were successfully retained for further analysis. We then proceeded with the downscaling clustering of these cells. Based on tissue-specific markers sourced from the relevant literature, these cells were classified into 8 major cell types, namely T_NK cells, B cells and plasma cells, myeloid cells, smooth muscle cells (SMCs), epithelial cells (EPCs), endothelial cells (ECs), Fibroblasts, and Pericytes (**Figure 1A**). For the BC patient cells belonging to these eight cell types, crucial characteristics such as G2M.score, S.score, nCount_RNA, and nFeature_RNA were visualized. **Figure 1B** illustrated the differential expression patterns of the top five marker genes across the eight distinct BC cell types. Through the analysis of cell type expression in the ER+ Tumor and Normal groups of BC, it was observed that the ER+ Tumor group had a significantly higher proportion of EPCs, whereas the Normal group had a greater prevalence of ECs and Fibroblasts (**Figure 1C**). **Figure 1D** provided a detailed illustration of the distribution of the ER+ Tumor group and the Normal group among the various cell types. Additionally, **Figure 1E** demonstrated the expression levels of different samples within the 8 different cell types. This comprehensive set of analyses and visualizations offers valuable insights into the cellular composition and characteristics of BC.

By employing inferCNV (**Supplementary Figure 1**) and subsequent dimensionality reduction clustering techniques, we were able to identify five distinct cell subpopulations. Based on differential gene expression analysis and specific biomarkers of each subgroup reported in the literature, five cell subgroups including were shown in **Figure 1F**: C0 *MUCL1*+ TCs, C1 *UGDH*+ TCs, C2 *ANKRD30A*+ TCs, C3 *PCLAF*+ TCs, and C4 *TM4SF1*+ TCs. We then analyzed the expression of these cell subpopulations in various samples within both the ER+ Tumor and Normal groups of BC. It was noted that the ER+ Tumor group showed a significantly greater percentage of C0 *MUCL1*+ TCs and C2 *ANKRD30A*+ TCs in comparison to the Normal group. On the contrary, the Normal group had a significantly higher percentage of C4 *TM4SF1*+ TCs than the ER+ Tumor group (**Figure 1G**). **Figure 1H** further illustrated the expression patterns of the five different cell subpopulations in both the ER+ Tumor and Normal groups, providing a more detailed understanding of the differences in cell subpopulation distribution and expression between these two groups.

**Supplementary Figure 2A** presented the expression levels of differential genes within the 5 cell subpopulations, offering insights into the unique genetic characteristics of each subgroup. The accuracy of the classification was verified by examining the expression of these marker genes in the corresponding cell populations. **Supplementary Figure 2B** provided a visual representation of the UMAP distribution of named genes across the five subpopulations, allowing for a better understanding of the spatial relationships between genes and cell types. **Supplementary Figure 2C** demonstrated that all five cellular subpopulations had a strong association with the Oxidative phosphorylation pathway among the metabolic pathways under analysis. This finding is significant as it indicates that oxidative phosphorylation may have a crucial role in the progression of many cancer cells, as supported by previous research (58). The correlation between the 5 cellular subpopulations and their highly expressed genes was also shown in **Supplementary Figure 2D**, highlighting the potential functional relationships between these genes and the cell subpopulations. Finally, **Supplementary Figure 2E** illustrated the expression of the identified genes within each respective cell subpopulation, providing a more detailed view of the gene expression patterns within each subgroup. Overall, these figures contribute to a more comprehensive understanding of the molecular and cellular characteristics of the studied cell subpopulations and their potential implications in cancer biology.

### Visualization of pseudotime series analysis of BC TCs subpopulations by CytoTRACE, monocle, and slingshot

**Figure 2:**
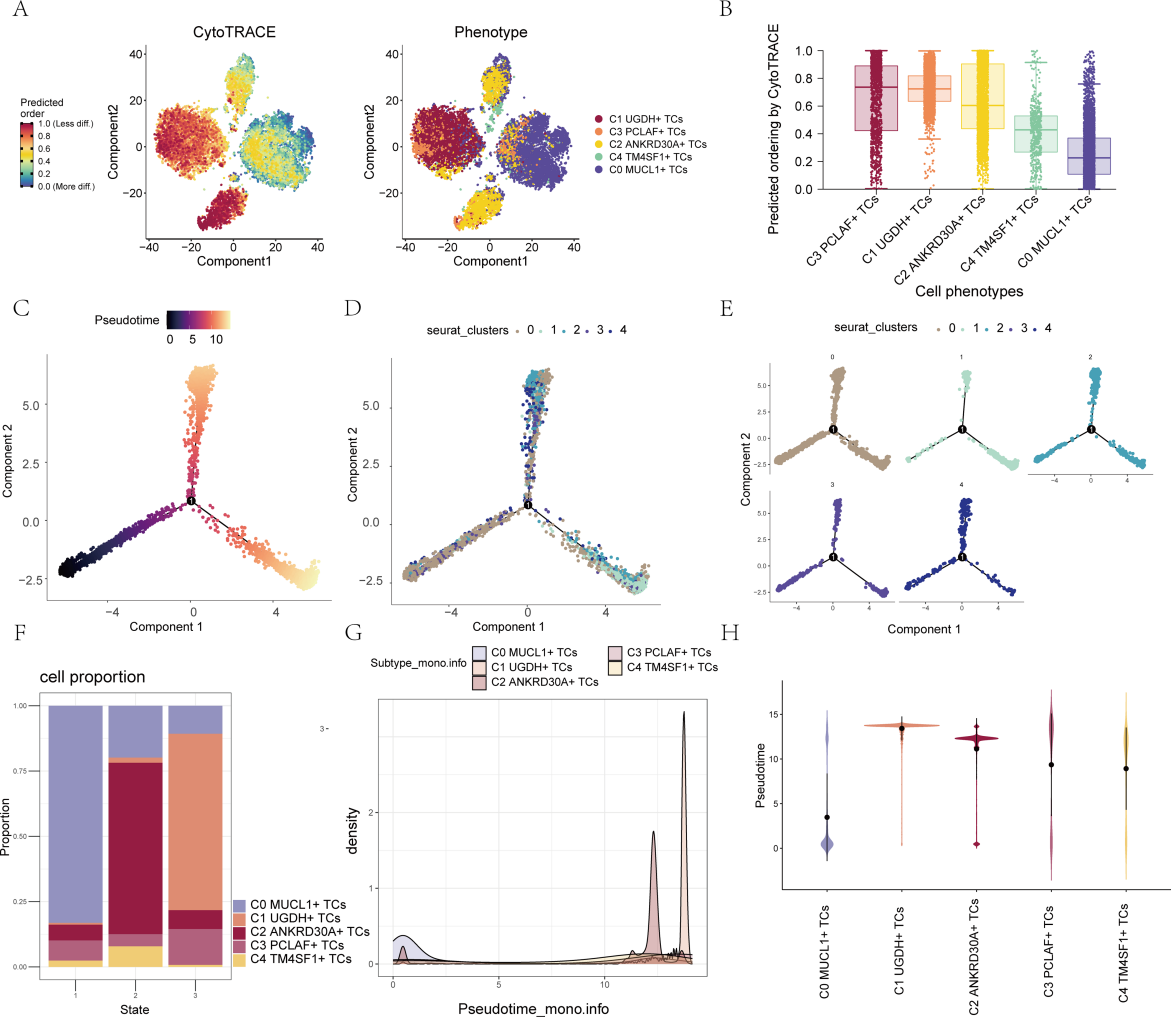
Visualization of pseudotime series analysis of BC TCs subpopulations by CytoTRACE, Monocle 2 and Slingshot. **(A)** The left figure represented the analysis of the differentiation of BC TCs using CytoTRACE and was shown in 2D. The color could represent the level of differentiation. The right figure represented the CytoTRACE results displayed according to different TCs subpopulations. The colors represented different TCs subpopulations. **(B)** Box line plot demonstrated the predicted ordering by CytoTRACE of TCs subpopulations. **(C)** UMAP plot demonstrated the differentiation of 5 TCs subpopulations on the pseudotime trajectory. **(D)** UMAP plot demonstrated cluster distribution of the 5 TCs subpopulations on the pseudotime trajectory. **(E)** Split-plane plot demonstrated the distribution of each of the 5 clusters on the pseudotime trajectory. **(F)** Bar graph demonstrated the expression in 5 different cell subpopulations in different states. **(G)** Ridge plot demonstrated the expression of the 5 cell subpopulations on the pseudotime. **(H)** Violin plot demonstrated the expression of 5 different cell subpopulations on the pseudotime.

In order to explore the differentiation and developmental associations among the five TCs subpopulations, CytoTRACE was employed to carry out the analysis and visualization of TCs differentiation, as depicted in **Figure 2A**. Box line plots were used to showcase the differentiation potential of these cell subpopulations. The results revealed that the cell stemness of the subpopulations decreased progressively in the order of C3 - C1 - C2 - C4 - C0, as illustrated in **Figure 2B**. Additionally, pseudotime analysis was performed to delve into the differentiation process of TCs during the development of cancer. This comprehensive set of analyses provided valuable insights into the dynamic changes and relationships within the TCs subpopulations during cancer progression.

UMAP plots were used to display the distribution of TCs clusters on the pseudotime sequence and the corresponding 5 states on the pseudotime differentiation trajectories, as shown in **Figures 2C, D**. Facet plots provided a detailed demonstration of the distribution position of cells in each cluster on the pseudotime series (**Figure 2E**). It was evident that the majority of C1 *UGDH*+ TCs were grouped at the end of the cell differentiation process. Bar graphs presented the distribution of the five cell subpopulations across the three states, with C0 *MUCL1*+ TCs being the most prevalent on state 1, C2 *ANKRD30A*+ TCs being the highest on state 2, and C1 *UGDH*+ TCs being the most abundant on state 3 (**Figure 2F**). Indeed, the ridge plot served as a crucial visual tool in this context. By clearly presenting the distribution of cellular subpopulations along the pseudotime, it lent further credence to the finding that C1 *UGDH*+ TCs occupies the terminal stage of pseudotime differentiation as seen in the facet plots (**Figure 2G**). The violin plot, on the other hand, focused on the expression levels of the five cell subpopulations throughout the pseudotime sequence. Its revelation that C1 *UGDH*+ TCs exhibited the highest expression levels at the end of pseudotime differentiation (**Figure 2H**) provided valuable information about the transcriptional activity of this particular subpopulation during the differentiation process. To deduce the continuous branching genealogical structure in TCs data, pseudotime trajectories of five cell subpopulations were analyzed using Slingshot, resulting in two lineages, Lineage1 and Lineage2. The distribution of these lineages was illustrated with a UMAP plot (**Supplementary Figure 3A**). Next, the relationship between the two Lineages and the pseudotime differentiation trajectories was shown (**Supplementary Figure 3B**), and it could be found that the starting positions of these two Lineages were the same, but the endpoints of the differentiation were different, with the endpoint of Lineage 1 being at C1 *UGDH*+ TCs, and the endpoint of Lineage 2 being at C3 *PCLAF*+ TCs. GO-BP enrichment was applied to analyze the two pseudotime trajectories. Analysis to visualize the two pseudotime trajectories, it was found that in the two trajectories, C1 was associated with biological processes such as differentiation, C3 was associated with biological processes such as cycle and mitotic, and C4 was associated with biological processes such as chemotaxis (**Supplementary Figure 3C**).

**Supplementary Figure 3D** provided scatter plots which served to depict the manner in which named genes were distributed among diverse subpopulations that were situated along Lineage 1 - 2. These scatter plots also effectively demonstrated the differentiation trajectories of the named genes over the course of pseudotime.

### CellChat analysis among all cells

**Figure 3:**
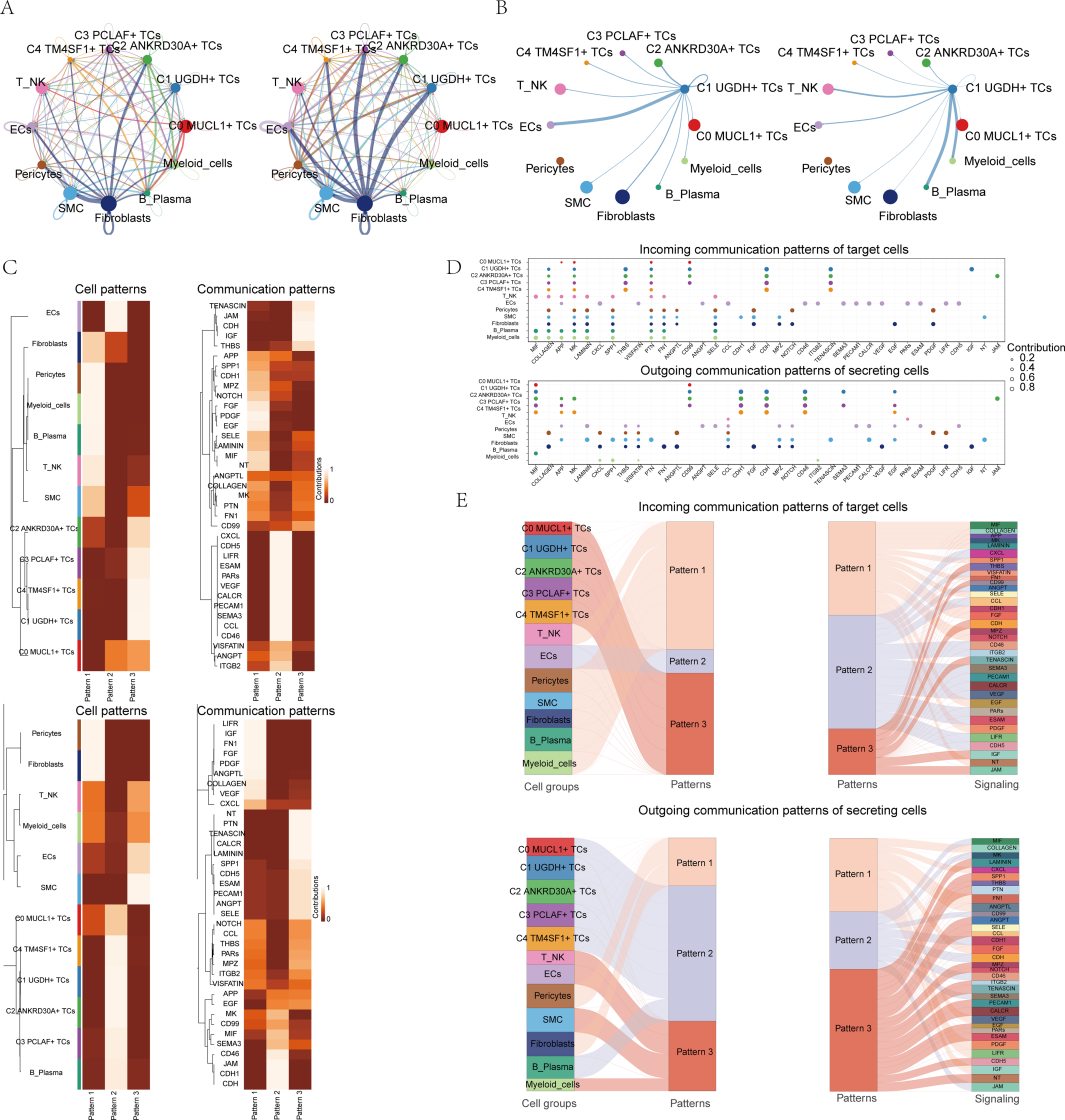
CellChat analysis between all cells. **(A)** Circle plots showed the number (left) and strength (right) of interactions between all cells. **(B)** String diagrams showed the correlation between C1 UGDH+ TCs and other cell subpopulations and cell types. **(C)** Heatmap showed pattern recognition of incoming cells (top), and outgoing cells (bottom) in all cells. **(D)** Outgoing contribution bubble plot and incoming contribution bubble plot showed cellular communication patterns between various cell subpopulations of TCs and other cells. **(E)** Sankey diagrams showed the inferred incoming and outgoing communication patterns of secretory cells, showing the correspondence between inferred potential patterns and cell populations, and signaling pathways. Top: Incoming Sankey diagram, bottom: Outgoing Sankey diagram.

In order to comprehensively and systematically decipher the complex responses that occur within cells, we embarked on an exploration of the relationships that exist between individual cells and the ligand-receptor communication networks. This was carried out with the intention of deepening our comprehension of the intricate web of cellular interactions. Through the utilization of CellChat analysis, we were able to construct elaborate intercellular communication networks that encompassed a wide range of cell types, including TCs, myeloid cells, Fibroblasts, and T_NK cells. Moreover, we were successful in quantifying the interactions that were manifested by the lines interconnecting the different cell types. The quantification of the interactions between cell types was achieved by taking into account both the number of pathways and the strength of the interaction. In **Figure 3A**, these two aspects were visually represented by the strength of the lines, where thicker lines were indicative of higher values in terms of both the number of pathways and the interaction strength. The circle plots presented in **Figure 3B** demonstrated the correlation between the quantity and intensity of interactions between C1 subpopulation and diverse cell types. It was evident that C1 exhibited a closer relationship with ECs, B cells and plasma cells, and myeloid cells.

We made use of CellChat’s gene expression pattern analysis methodologies to conduct an in-depth examination of cell and signaling pathway interactions. At the outset, we determined the connection between the inferred potential communication patterns and the secreted cell groups, with the aim of grasping the efferent communication patterns. Three distinct incoming signaling patterns were discerned: pattern 1, which consisted of C0 *MUCL1*+ TCs, C1 *UGDH*+ TCs, C3 *PCLAF*+ TCs, C4 *TM4SF1*+ TCs, and ECs; pattern 2, encompassing Pericytes, myeloid_cells, B cells and plasma cells, T_NK cells, SMC, and C2 *ANKRD30A*+ TCs; and pattern 3, involving ECs, Fibroblasts, Pericytes, myeloid_cells, B cells and plasma cells. In the similar circumstance, three outgoing signaling patterns were detected: pattern 1, composed of C1 *UGDH*+ TCs, C2 *ANKRD30A*+ TCs, C4 *TM4SF1*+ TCs, and SMC; pattern 2, including Pericytes, Fibroblasts, T_NK cells, and SMC; and pattern 3, made up of Pericytes, Fibroblasts, C0 *MUCL1*+ TCs, C1 *UGDH*+ TCs, C2 *ANKRD30A*+ TCs, C3 *PCLAF*+ TCs, and C4 *TM4SF1*+ TCs. Each of these patterns was associated with specific incoming and outgoing signaling, as depicted in **Figure 3C**. In the context of BC, every cell type is capable of serving as both a signal transmitter and a receiver, and the ligand-receptor interactions among these cell types contribute to the development of BC, as illustrated in **Figure 3D**.

In addition to the investigation of communications within individual pathways, a crucial question that arose was regarding the manner in which diverse cell populations and signaling pathways manage to coordinate their respective functions. To address this, CellChat made use of a pattern recognition approach that was based on nonnegative matrix decomposition. This approach was specifically designed to detect global communication patterns and to pinpoint the key signals present across a variety of cell groups.

Upon the application of this particular analysis, it became evident that there were three distinct outgoing signaling patterns as well as three incoming signaling patterns. The outcomes of this analysis further demonstrated that the incoming signaling directed towards the subpopulations of TCs mainly adheres to pattern 3. This pattern 3 was found to encompass a range of communicating molecules, including but not limited to CD99, JAM, and CDH.

On the contrary, an examination of the communication patterns of the target cells brought to light that the outgoing signaling of TCs was predominantly regulated by pattern 2. This pattern 2 encompassed several signaling pathways such as MIF, CD99, and CDH1, along with others, as can be seen in **Figure 3E**. Ultimately, our investigation led to the discovery that CDH was intimately associated with the subpopulations of TCs in both the incoming and outgoing aspects, as depicted in **Supplementary Figure 4A**. CDH (Cadherin) family is an important cell adhesion molecule, which plays a key role in the occurrence, development, invasion and metastasis of breast cancer

To gain a deeper understanding of the CDH signaling pathway and its associated pathways, a comprehensive visualization and analysis were carried out. Cell types were carefully identified as both mediators and influencers in the context of CDH signaling-mediated intercellular communication. Among the TCs subpopulations, it was observed that C1 *UGDH*+ TCs displayed the highest level of expression within the CDH signaling pathway, as clearly shown in **Supplementary Figure 4C**. A slice map was presented, which effectively depicted the targeting of CDH released by all cell types, as illustrated in **Supplementary Figure 4B**. Additionally, a violin plot was employed to vividly demonstrate that the TCs subpopulation C1 *UGDH*+ TCs exhibited a notably high expression of *CDH1*, a gene that is closely related to the CDH pathway, when compared across different cell types, as can be seen in **Supplementary Figure 4D**.

Subsequently, the correlation between various cell types within the CDH signaling network was further elucidated through the use of heatmaps, as presented in **Supplementary Figure 4E**.

### Screened the genes that made up the risk score group and conducted an association analysis

**Figure 4:**
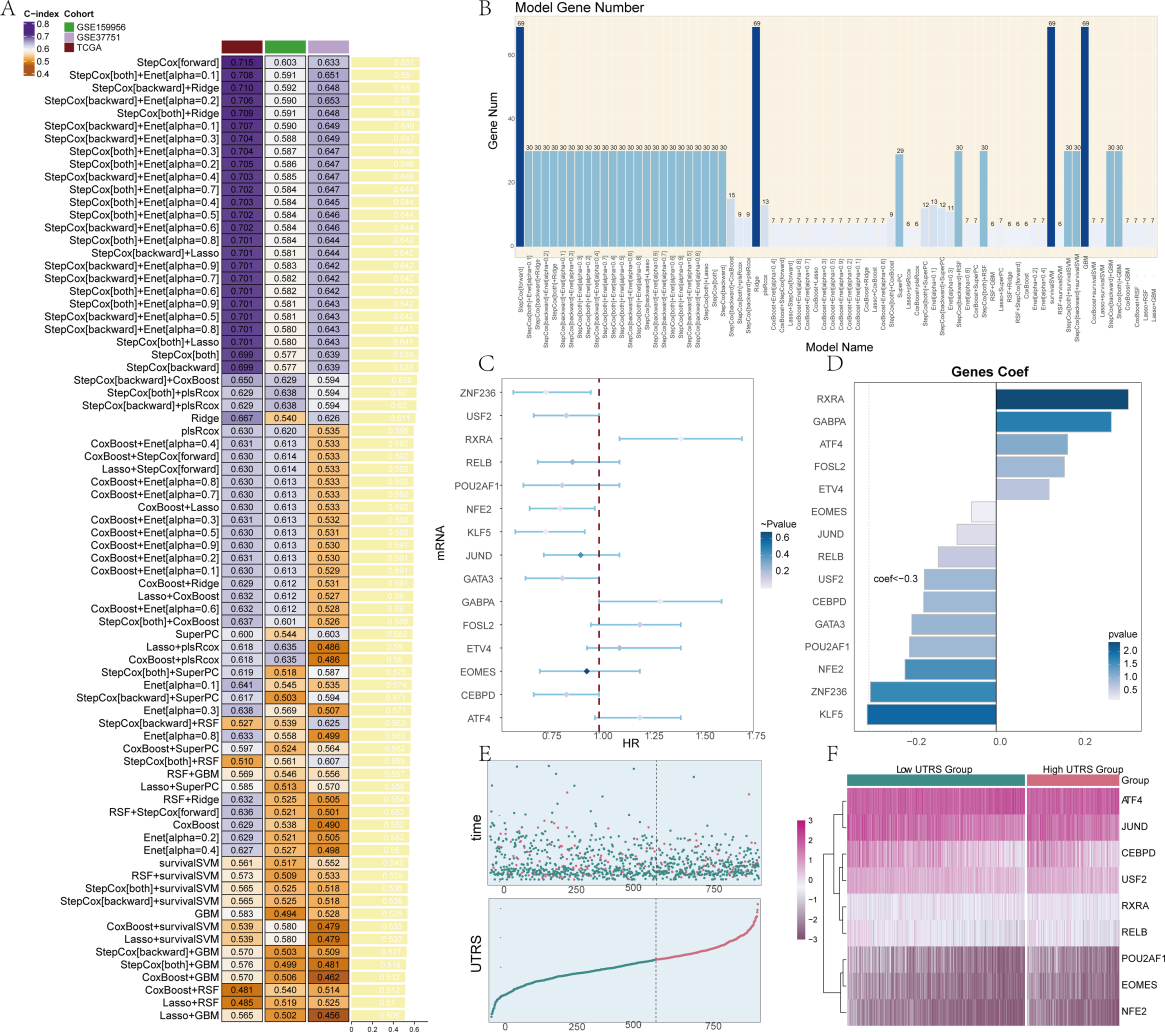
Construction of prognosis model and survival analysis. **(A)** A total of 101 prediction models were cross-validated by the ten-fold framework, and the C-index of each model was further calculated across all validation datasets. **(B)** We screened 15 model genes using StepCox[backward]+CoxBoost. **(C)** The forest map showed a Univariate analysis of the Risk score genes. HR<1 protective factors, HR>1 risk factors. **(D)** The bar chart displayed the expression levels of 15 genes when the coefficient value for the genes was set as 0, providing information about the relative expression levels of these genes within the context of the analysis. **(E)** The scatter plot and curve plot depicted the risk scores of two groups: high and low UTRS groups, allowing for a comparison of the risk scores between these two groups, and the curve plot showed how the UTRS change over a specific range or threshold. **(F)** The heatmap represented the differences in gene expression between the high and low UTRS groups, using color scales based on standardized data. The low Risk score group was represented by green, while the high UTRS group was represented by red.

We conducted a meticulous cross-checking process on a total of 101 prediction models with the aim of obtaining the C-index for each individual model. Through this comprehensive analysis, we were able to identify and obtain 15 genes that are crucial for building models by utilizing the StepCox[backward]+CoxBoost method. The details regarding this process can be further visualized and understood through **Figures 4A, B**.

In the course of this particular work, a detailed examination of the clinical significance pertaining to the identified cell types was carried out. This was achieved by performing a univariate COX analysis specifically on the top 100 marker genes of C1 UGDH+ TCs. The results of this analysis brought to light that a total of 15 genes were found to be associated with patient prognosis. These genes included Z*NF236, USF2, RXRA, RELB, POU2AF1, NFE2, KLF5, JUND, GATA3, GABPA, FOSL2, ETV4, EOMES, CEBPD*, and *ATF4*, as clearly depicted in **Figure 4C**. Among these 15 genes, the majority of them were regarded as protective variables. However, a small percentage of these genes were identified as risk factors, with the significance level being set at P < 0.05. To further illustrate the situation, a bar chart was generated. This bar chart was designed to display the expression of the 15 genes. In the process of creating this bar chart, the gene coef value was set to 0, and 0 was taken as the standard for comparison, as shown in **Figure 4D**.

The risk score for each patient within the TCGA-BRCA dataset was computed by making use of the expression levels of the 15 genes along with their respective regression coefficients. Subsequently, the distribution of these risk scores within the TCGA- BRCA cohort was presented. The patients were categorized into two distinct groups, namely the high and low UTRS (*UGDH* tumor risk score) groups, based on the optimal cutoff value. Both the scatter plot and the curve plot were utilized to illustrate the risk scores of these two groups, namely the high and low UTRS groups. This allowed for a direct comparison of the risk scores between the two groups. Moreover, the curve plot also demonstrated how the risk scores vary over a particular range or threshold, as can be seen in **Figure 4E**. In order to investigate the differences in gene expression levels that exist between the two groups, a heatmap was employed. This heatmap effectively represented the disparities in gene expression between the high and low UTRS groups, as depicted in **Figure 4F**.

Subsequently, a survival analysis was conducted by us. In this process, the 15 selected risk score genes were divided into two groups, namely the high UTRS group and the low UTRS group. It was observed that the group with a high risk score demonstrated a significantly worse prognosis when compared to the group with a low risk score, as illustrated in **Supplementary Figure 5A**. Additionally, the profile of each gene along with its associated prognosis is presented in **Supplementary Figure 6**. To evaluate the independence of risk variables, a gene-cell clinical prediction model was constructed. This was achieved by combining various clinical pathological aspects, such as age, race, T, N, and M stages, with the high and low UTRS groups through the application of multivariate Cox regression. The analysis outcomes indicated that the UTRS served as a distinct and unique risk factor, as shown in **Supplementary Figure 5B**.

A nomogram chart was then generated with the aim of displaying the survival rates after one, three, and five years. This nomogram incorporated multiple factors including age, race, T, N, and M stages, as depicted in **Supplementary Figure 5C**. Furthermore, the survival graphs and ROC curves of the two data sets GSE37751 and GSE159956 were also presented (**Supplementary Figure 7**). It was noted that the survival conditions within the high UTRS group were also considerably worse in these data sets.

### Comparative analysis of immune infiltration between high and low UTRS groups

**Figure 5:**
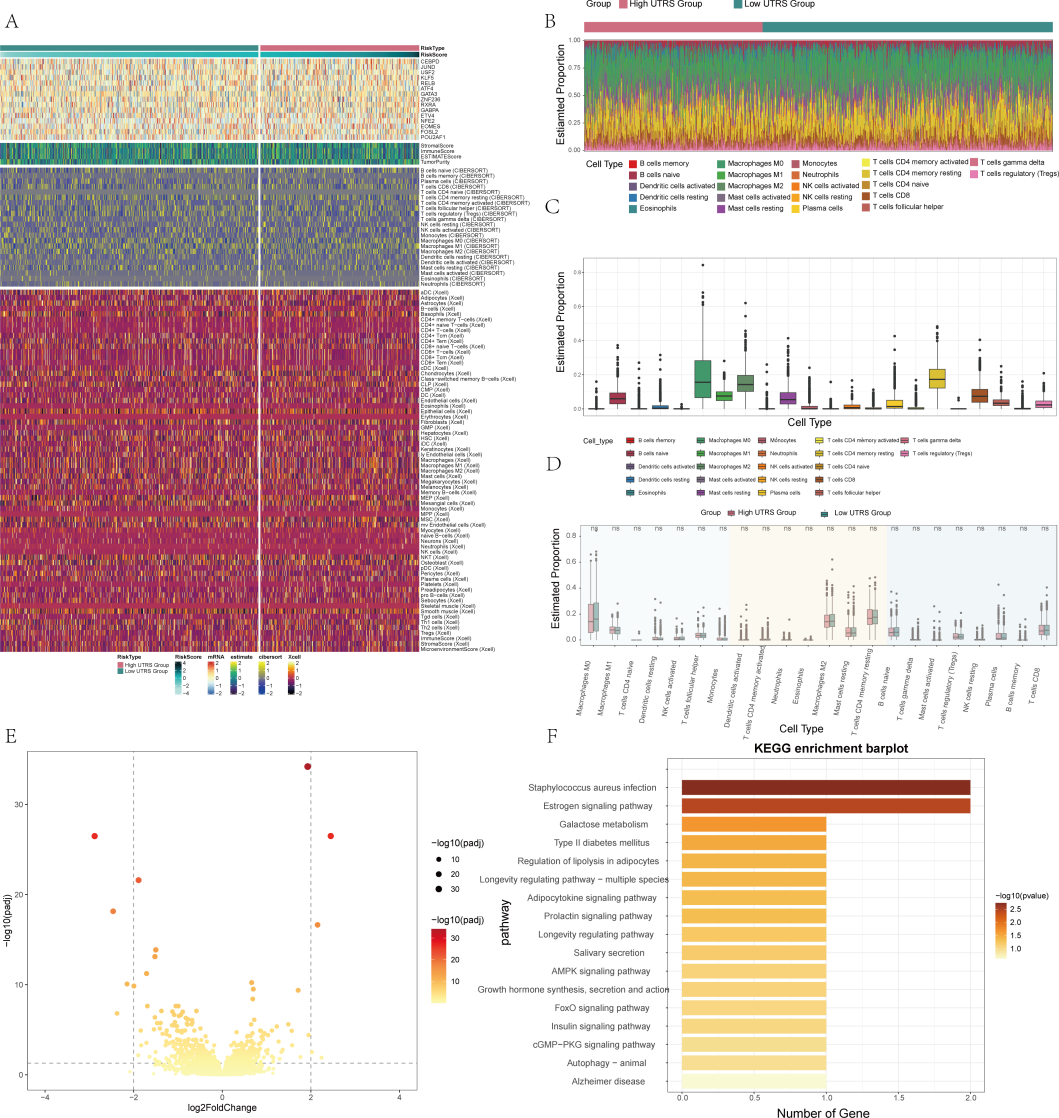
Differential analysis of immune infiltration in high and low risk score groups. **(A)** Heatmap showed differential expression in the immune infiltrates of high and low risk score group. **(B)** Stacked bar graph of immune infiltration. **(C)** Estimated proportion of 22 immune cells was shown by box-and-line plot. **(D)** Eestimated proportion of 22 immune cells in high and low risk score groups was shown by box-and-line plot. **(E)** Volcano plot showed DEGs in high and low risk score groups. **(F)** Results of enrichment on different pathways was shown by KEGG enrichment analysis of differential genes.

In the course of this study, an in-depth examination was carried out to explore the differences in immune infiltration between the high and low UTRS groups. The focus was on analyzing the association that exists between immune cells and these respective groups. A heatmap was employed to vividly illustrate the differential expression of scores and cells by making use of the ESTIMATE, CIBERSORT, and XCELL methods, as clearly depicted in **Figure 5A**. We further utilized stacked bar graphs to effectively illustrate the predicted abundance of various immune cells, thereby providing a clear showcase of the immune infiltration situation, as presented in **Figure 5B**. Additionally, box line plots were used to illustrate the estimated proportions of various immune cells. Through this analysis, it was revealed that T cells CD4 memory resting exhibited the highest expression level, as can be seen in **Figure 5C**. Moreover, by applying the CIBERSORT algorithm, we conducted an analysis of immune cell infiltration in BC patients sourced from the TCGA database. This analysis successfully revealed the predicted abundance of immune cells in both the high and low UTRS groups, as shown in **Figure 5D**.

### Variance and mutation analysis were used to analyze the differences between the two groups

In order to delve into the distinctions between the high and low UTRS groups, a comprehensive set of analyses was carried out. Volcano plots, as depicted in **Figure 5E**, were utilized to vividly illustrate the differential gene expression that existed between these two groups. To gain a clearer understanding of the potential functions and roles that each subpopulation might play in the initiation and progression of BC, both functional enrichment and KEGG enrichment analyses were conducted. The outcomes of these analyses for different genes were presented in the form of bar charts. The findings from these investigations revealed that the differential genes were predominantly associated with several key aspects, including Staphylococcus aureus infection, the Estrogen signaling pathway, and a number of other relevant pathways, as shown in **Figure 5F**. Furthermore, through high-throughput mRNA sequencing, in-depth bioinformatics analysis, and pharmacological studies, it was uncovered that Staphylococcus aureus has the ability to facilitate breast cell metastasis. This occurs specifically through the innate immune pathway, especially within cancer cells (59). Estrogens play an important role in regulating the growth and differentiation of normal, premalignant and malignant cell types, especially breast epithelial cells (60).

The GOBP enrichment analysis of the differential genes provided valuable insights into their participation in specific pathways. As demonstrated by the dot graphs in **Supplementary Figure 8A**, these differential genes were found to be involved in several key pathways. These included intermediate filament organization, which pertains to the structuring and arrangement of intermediate filaments within cells. Another pathway was body fluid secretion, which is crucial for the proper functioning of various physiological processes involving the release of fluids. Additionally, intermediate filament cytoskeleton organization was also implicated, highlighting the role of these genes in maintaining the integrity and functionality of the intermediate filament cytoskeleton. Moreover, intermediate filament-based processes were also part of the pathways associated with these differential genes. Furthermore, the GSEA scoring of the GO-BP enrichment items for the differential genes shed light on the enrichment scores across different pathways. This information, as presented in **Supplementary Figure 8B**, allowed for a more detailed understanding of the significance and relative importance of the enrichment of these genes within the various pathways under consideration.

**Supplementary Figure 8C** provided a visual representation of the correlation among the mutations of genes that contribute to the UTRS score in the thermogram. To further explore the correlation between gene mutations and the immune components within the TME, additional research was conducted. In the initial stage, the top 20 most frequently mutated genes were identified within two somatic cell groups. The upper bar graph was utilized to depict the mutation load on a per-sample basis, while the right bar graph was employed to illustrate the overall mutation ratio of each gene across these samples, as shown in **Supplementary Figure 8D**. By employing histograms to display chromosome gain and loss, it was evident that the highest level of CNV gain was observed in *CEBPD*, whereas the maximum CNV loss occurred in *POU2AF1*, as depicted in **Supplementary Figure 8E**. The analysis and visualization of cell mutation data from both groups led to the discovery of mutations in 15 genes within the model, as illustrated in **Supplementary Figure 8F**.

We made use of various graphical representations such as box charts and bar charts to showcase the overall mutation situation in patients with TCGA- BRCA. Specifically, in **Supplementary Figure 8G**, the box chart and percentage histogram were utilized to present the mutation status. The box chart located at the top left displayed the total mutation situation of all samples. The box diagram at the top right illustrated the ratio of base transversion and transformation, where Ti denoted transformation (involving the replacement of purine by purine and pyrimidine by pyrimidine) and Tv denoted transversion (substitution between purine and pyrimidine). The percentage bar chart positioned below showed the mutation details in each sample, as presented in **Supplementary Figure 8H**.

### Identified TFs regulator modules driving BC cell subset functions

**Figure 6:**
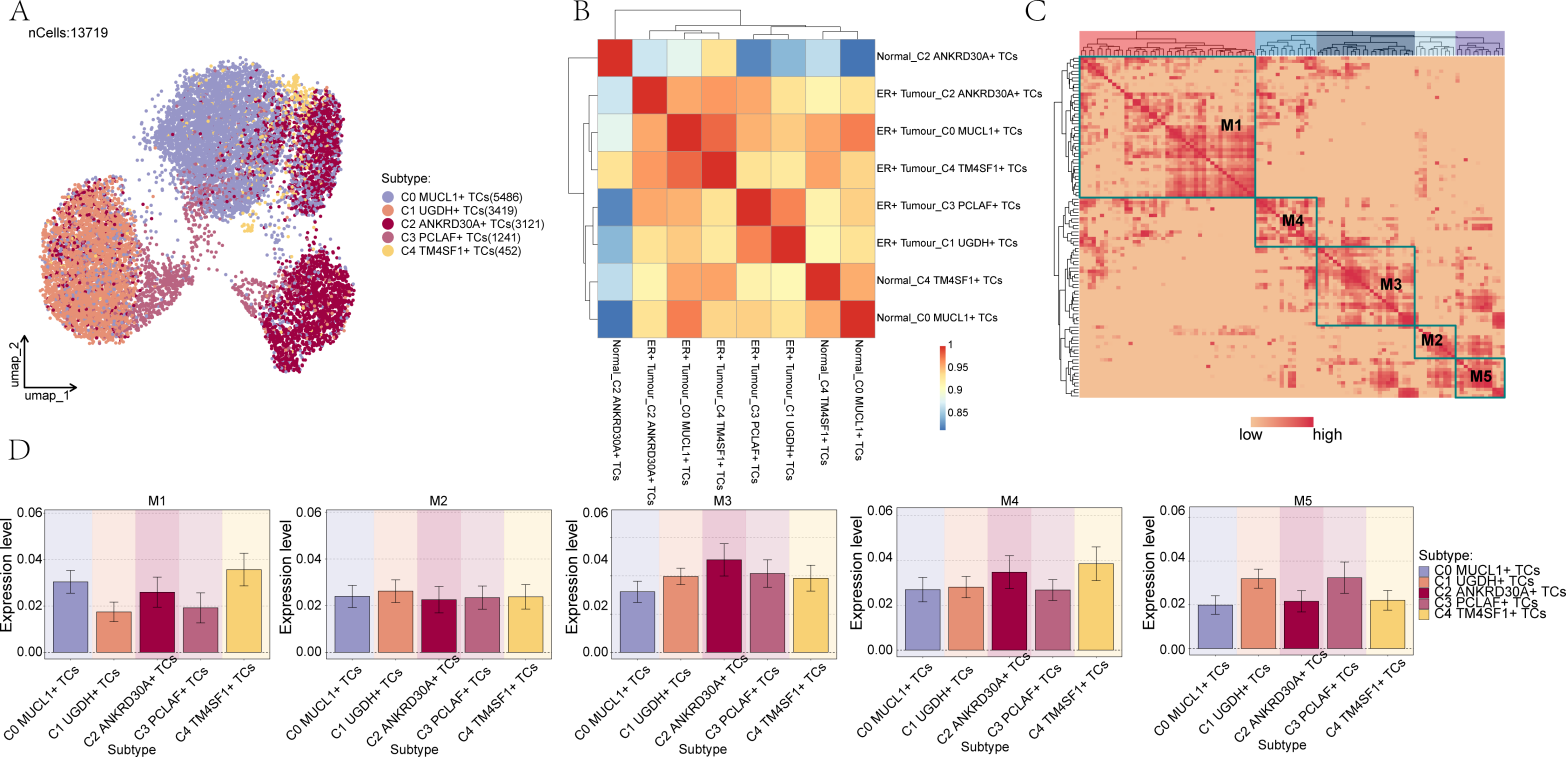
Identification of TF regulator modules in BC cell subpopulations. **(A)** UMAP plot demonstrated the distribution of the 5 cell subpopulations in BC. **(B)** Heatmap demonstrated the connection between the 5 cellular subpopulations and subpopulations in BC cells. **(C)** Identification of 5 regulatory submodules of BC cell subpopulations based on the CSI substrate. **(D)** Box line of the expression of the 5 TF regulatory submodules of BC cell subpopulations.

To identify the core TFs within BC cell subpopulations, SCENIC analysis was carried out. PySCENIC, in particular, has the capability to infer gene regulatory networks that span across all BC cell subpopulations. Based on the UMAP plot of BC subpopulations, as depicted in **Figure 6A**, the relationship between the five cellular subpopulations of BC cells and their respective phases was demonstrated in combination with a heat map, as shown in **Figure 6B**. Furthermore, through the utilization of the connectivity specificity index (CSI) substrate, we were able to identify five regulatory submodules of BC cell subpopulations. These submodules were divided into five primary modules, namely M1, M2, M3, M4, and M5, as illustrated in **Figure 6C**.

To improve the visualization of gene expression, bar graphs and scatter plots were utilized. Bar graphs and scatter plots were employed to illustrate the expression of BC cell subpopulations with regard to the five TF regulator modules, as presented in **Figure 6D** and **Supplementary Figure 9A**. Moreover, scatter plots were used to depict the fraction of variance across different subpopulations and groups respectively, as shown in **Supplementary Figures 9B, C**.

**Supplementary Figure 9D** presented the ranking of regulons within BC cell subpopulations based on their specificity scores. It was evident that among the transcription factors corresponding to the C1 subpopulation, *CEBPD* exhibited the highest specificity score.

### Experimental verification has proved that knocking down CEBPD can affect the proliferation and migration of tumor cells

**Figure 7:**
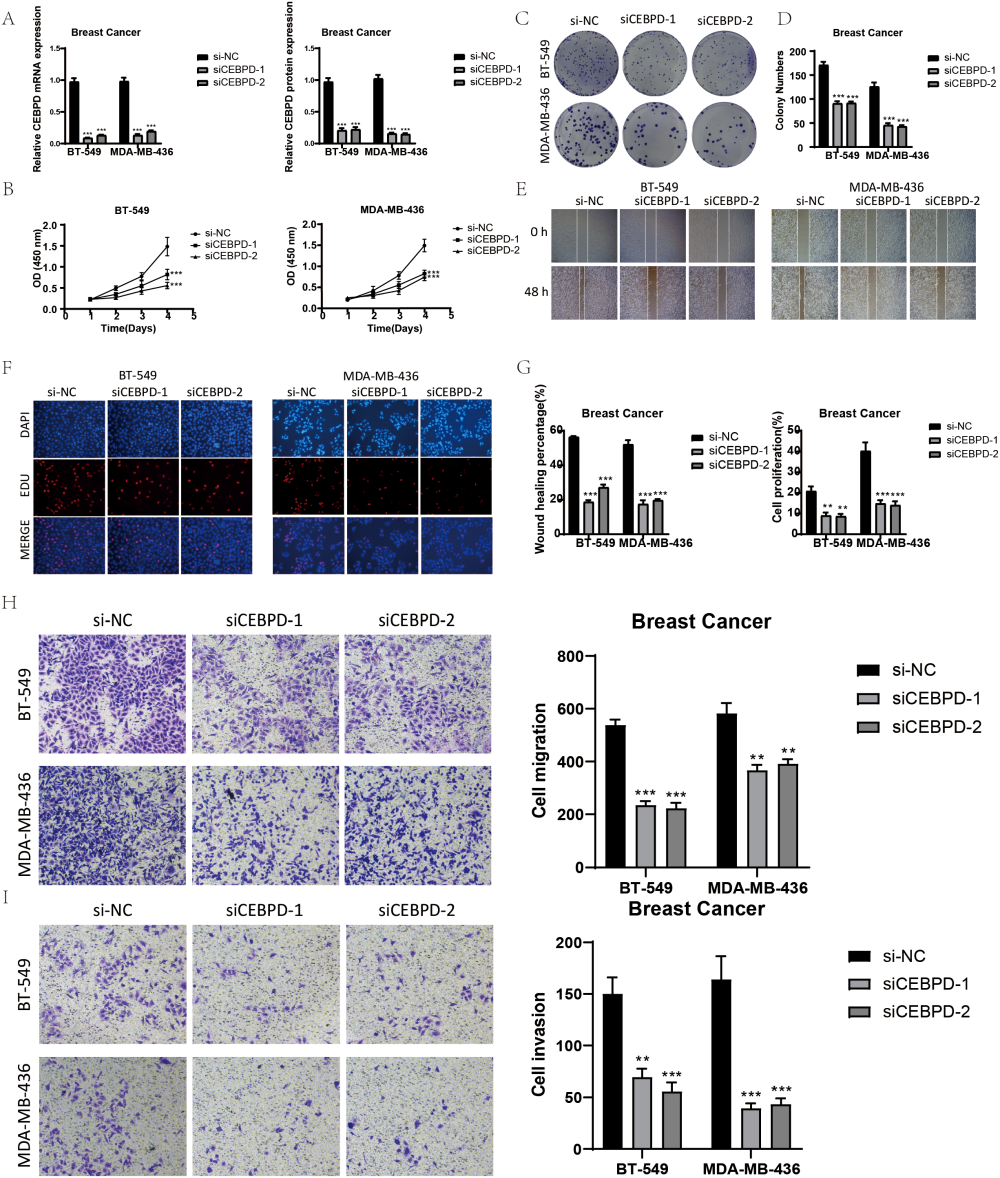
*In vitro* experimental validation of CEBPD. **(A)** After CEBPD knock-down, the expression levels of mRNA and protein decreased significantly. **(B)** CCK-8 detection showed that compared with the control group, the cell viability was significantly decreased after CEBPD knock-down. **(C, D)** The colony formation assay showed that the number of colonies decreased significantly after CEBPD was knocked down. **(E)** Scratch test showed that CEBPD knockdown inhibited cell migration. **(F)** EDU staining confirmed that CEBPD knock-down had inhibitory effect on cell proliferation. **(G)** Bar chart showed that the ability of cell migration and proliferation decreased significantly after CEBPD. **(H, I)** Transwell experiment showed that CEBPD knockdown inhibited the migration and invasion of TCs in BT-549 and MDA-MB-436 cell lines. *, p < 0.05; **, p < 0.0 1; ***, p < 0.001 indicates a significant difference, and NS indicates a non-significant difference.

In order to conduct a more in-depth exploration of the role played by *CEBPD*, we carried out *in vitro* experiments by utilizing the BT-549 and MDA-MB-436 cell lines (61). Initially, we proceeded with the knockdown of *CEBPD*. Subsequently, we measured the amounts of mRNA and protein expression both in the pre-knockdown and post-knockdown states. It was discovered that in both of these cell lines, the levels of mRNA and protein expression were substantially lower when compared to those of the control groups, as clearly demonstrated in **Figure 7A**. After the *CEBPD* knockdown, the results of the CCK-8 test indicated a significant decline in cell viability, as shown in **Figure 7B**.

The EDU and colony formation assays were then employed to verify the impact of *CEBPD* knockdown. These assays effectively demonstrated that the knockdown of *CEBPD* prevented cell division, as depicted in **Figures 7C, D, F, G**.

Moreover, according to the scratch and transwell experiments, the knockdown of *CEBPD* led to a dramatic decrease in cell migration and invasion, as presented in **Figures 7E, G–I**.

Collectively, these findings strongly suggested that the knockdown of CEBPD had the effect of suppressing tumor development by impeding cell activity, proliferation, migration, and invasion.

## Discussion

The development and application of scRNA-seq within the realm of cancer research have significantly enhanced the diagnosis, treatment, and prognosis prediction of numerous malignancies. This has been achieved by delving deeper into our understanding of the biological characteristics and dynamic processes that occur within malignant regions, as evidenced by previous studies (62–64). In this research endeavor, a meticulous examination was carried out on the tumor epithelial cells present within BC. The objective was to confirm the pro-oncogenic role played by a specific subpopulation. This was achieved through a comprehensive analysis of C1 *UGDH*+ TCs along with their various subpopulations.

Subsequently, the transcription factor *CEBPD* was successfully identified by means of cellular communication analysis. To further solidify the understanding of its function, the pro-oncogenic role of this gene was rigorously validated through the implementation of a cellular knockdown assay. This involved manipulating the cellular environment to observe the effects of reducing the expression of *CEBPD* and thereby determining its impact on oncogenic processes (65).

This study harnessed the power of scRNA-seq technology to conduct an in-depth and comprehensive characterization of the cellular heterogeneity exhibited by TCs. Through this analysis, we were able to identify a diverse range of cell types. Among the non-immune cells, smooth muscle cells were detected, while in the realm of immune cells, T_NK cells, MCs, and myeloid cells were discovered. Moreover, we undertook a detailed and meticulous analysis of the sample sources from which these various cell types were derived, as well as their distribution characteristics during that particular stage of the study. Among these cell types, EPCs particularly caught our attention. It is well-known that EMT plays a crucial role in the progression of early-stage tumors, transforming them into more aggressive malignancies, as described in reference (66). This process is inextricably linked to the formation of BC. Tumor invasion and metastasis are indeed the primary factors contributing to recurrence and mortality following treatment in BC patients. This fact underlines the significance of thoroughly describing and analyzing the TME with specific regard to EPCs. In prior studies, as indicated by references (44, 67), EPCs have been demonstrated to be associated with pro-tumorigenic effects, further emphasizing the importance of understanding their role within the context of BC and the TME.

Although there is an increasing body of evidence regarding the presence of EPCs within tumors, the exact role that these EPCs play within the TME remains to be fully elucidated (44). Consequently, our research efforts were directed towards a detailed examination of EPCs. To achieve this, we employed the inferCNV technique followed by dimensionality reduction clustering. Through this approach, we were able to successfully identify five distinct cell subpopulations.

By integrating the information about the sample sources, it was observed that the cell subpopulation C1 only emerged within the ER+ Tumor group. Notably, the ER+ tumor group demonstrated a considerably higher abundance of C1 *UGDH*+ TCs in comparison to the Normal group. This disparity can likely be attributed to the role that ER plays. ER is involved in promoting the proliferation and expansion of breast epithelial cells. Such actions have a significant impact on various aspects of breast health, including breast development, the process of carcinogenesis, and the progression towards more severe systemic diseases (68). By examining the top 5 metabolism-related pathways of each subpopulation, it was determined that oxidative phosphorylation exhibited the highest correlation with the C1 subpopulation. There is existing evidence suggesting that oxidative phosphorylation plays a crucial role in the progression of numerous cancer cells (58). From the perspective of cell proliferation, breast cancer cells often require more energy than normal cells to maintain their rapid division and growth. Oxidative phosphorylation pathway can produce ATP efficiently, providing sufficient energy for the energy-consuming processes of DNA replication, protein synthesis and cell division of breast cancer cells. In some breast cancer subtypes, such as C1 UGDH + subgroup, the activity of oxidative phosphorylation pathway is significantly enhanced, which accelerates the proliferation rate of these cells and promotes the growth and development of tumors. In the predicted ordering based on CytoTRACE, the C1 *UGDH*+ TCs were expressed at relatively higher levels, which implies a greater degree of differentiation. This, in turn, indicates a higher level of cell stemness within this subpopulation (69). During the pseudotime analysis, the C1 *UGDH*+ TCs subpopulation was predominantly observed at the terminal stage of the differentiation trajectory. This positioning suggests a higher level of malignancy. Moreover, the high stemness of this cell subpopulation is closely associated with the tumor metastatic ability (70). Finally, in the slingshot analysis, the subpopulation of C1 *UGDH*+ TCs was located at the end of Lineage1, which is equivalent to the end of the differentiation process. This finding further validates the aforementioned points.

Consequently, the target subpopulation was identified as C1 *UGDH*+TC through the identification of highly expressed genes in each subpopulation along with the slingshot pseudotime analysis.

We made use of CellChat communication pattern analysis to delve into the interactions that occur between the C1 *UGDH*+ TCs subpopulation and other cell types. Through the application of CellChat analysis, we were able to construct intercellular communication networks. These networks involved T_NK cells, B cells and plasma cells, myeloid cells, as well as various TC subpopulations, with the aim of clarifying the interactions between the C1 *UGDH*+ TC subpopulations and other cell types. Furthermore, we identified three efferent and afferent patterns, along with their corresponding signaling pathway expressions. As a result of this analysis, we discovered the CDH signaling pathway. It was found that C1 is highly expressed on CDH. Moreover, it has been confirmed in previous studies that several CDH-related genes play a role in promoting tumor growth. Based on these findings, it can be inferred that the C1 subpopulation may also have a role in promoting tumor development (71).

Immune cells serve as the cornerstone of immunotherapy. Understanding immune infiltration is of utmost importance as it is crucial for uncovering molecular mechanisms and for the development of novel strategies to improve clinical outcomes (72). Consequently, with the aim of further exploring the roles of two distinct subpopulations in tumor progression, we focused our study on the high UTRS group and the low UTRS group. We then proceeded to analyze the immune infiltration within these two groups. It was observed that the high UTRS group was predominantly composed of Macrophages M1. In contrast, the low UTRS group encompassed various cell types such as Macrophages M0, Macrophages M2, resting memory CD4 T cells, and naive B cells (73). The survival rate of the low UTRS group was found to be better, whereas the survival rate of the high UTRS group was poorer. This indicates that the low UTRS group may potentially benefit from immunotherapy, while the high UTRS group may exhibit resistance to it. To investigate the impact of the C1 UGDH+ TCs subpopulation on tumors, an enrichment analysis was conducted. This analysis identified specific genes such as KRT13, UPK1B, and FTHL17. KRT13 has the potential to promote tumor metastasis (74). UPK1B, which is a transmembrane tetraprotein, is associated with the tumorigenesis and progression of bladder, stomach, and colorectal cancers. FTHL17 is linked to cancer development, particularly in colon cancer (75). The enrichment pathway analysis using GO-BP and KEGG for the C1 *UGDH*+ TCs subpopulation revealed its extensive involvement in pathways such as intermediate filament organization, body fluid secretion, and processes related to the intermediate filament cytoskeleton. All of these pathways suggest that the C1 *UGDH*+ TCs subpopulation may be involved in EMT.

In summary, based on the up-regulated genes and the enrichment pathways identified, we propose that the C1 *UGDH*+ TCs subpopulation represents TCs within epithelial cells. These cells display a loss of apical polarity and adhesion, while simultaneously acquiring mesenchymal characteristics and migration capabilities. This, in turn, promotes tumor progression and enhances the tumorigenic, metastatic, and drug resistance properties of cancer cells (76, 77).

To investigate the relationship between this subpopulation and prognosis, we established a prognostic score (UTRS) to evaluate its relationship with prognosis. It was found that the prognosis of patients in the high-risk group was significantly worse than that in the low-risk group, indicating that the tumor cells in the high-risk group may have more characteristics of tumor stem cells, and may be related to the stronger proliferation and invasion ability of tumor cells, which is reflected in the context of the discussion. Compared to other models, our model shows higher predictive accuracy. By calculating the area under the curve (AUC) to evaluate the model’s ability to distinguish the prognosis of breast cancer patients, our model can predict the prognosis of patients more accurately, and provide a more reliable decision basis for clinicians. In addition, our risk score model is based on a few easy-to-obtain clinical and molecular indicators, which is more convenient for clinical application than other models that use a large number of complex detection indicators. Breast cancer is highly heterogeneous, and the tumor cells of different patients have great differences in gene expression and molecular characteristics. Our model can better capture this tumor heterogeneity and more comprehensively characterize tumor cells through comprehensive analysis of single-cell data or multi-omics data, thereby improving prognosis prediction accuracy for patients with different subtypes of breast cancer. In contrast, some traditional models may not fully consider tumor heterogeneity, resulting in inaccurate prognosis assessment for certain subtypes of patients (78).

The gene regulatory network of C1 UGDH+ TCs was analyzed through the application of pySCENIC, which led to the identification of *CEBPD* as a key regulatory factor. Analyses of clinical samples and public databases demonstrated that *CEBPD* was significantly up-regulated in glioblastoma. Moreover, elevated levels of *CEBPD* were associated with a poor prognosis. Additionally, under anoxic conditions, *CEBPD* was highly expressed in glioblastoma tissues and cell lines (79). These findings suggest that *CEBPD* may also play a role in promoting the development of BC.

Simultaneously, to further verify the role of *CEBPD* in BC, *in vitro* experiments were carried out using the BT-549 and MDA-MB-436 cell lines. Through these experiments, it was observed that the knockout of *CEBPD* inhibited the activity, migration, and proliferation of TCs, thereby suppressing tumor growth. This effectively verified the conjecture that *CEBPD* could promote the development of BC.

Our study was designed with the aim of highlighting the role of TCs in the progression of BC, addressing related concerns, and enhancing the understanding of the BC tumor microenvironment. The novelty of this study lies in its exploration of BC from a new perspective. It provides a fresh outlook on targeting EPCs for BC treatment and supports *CEBPD* as a potential cancer therapeutic target. Moreover, we were able to discover the communication pathways between tumors and our target TCs subpopulations.

It is anticipated that the development of targeted therapies against *UGDH+ TCs* will progress further in the future. In the future, we will carry out research on UGDH+ TCs from many aspects, such as: To study which transcription factors directly regulate the expression of UGDH subsets related genes, and identify the binding sites between transcription factors and UGDH gene promoter or enhancer regions by ChIP-seq (chromatin immunoprecipitation sequencing) and other technologies, so as to clarify the transcriptional regulatory network. To explore the synergistic relationship between UGDH subgroup and other cell subgroups in metabolism; Explore the use of UGDH subgroups in combination with other known disease markers to improve the sensitivity and specificity of diagnosis (80). Patients with different UTRS levels were associated with clinicopathological characteristics of tumors, such as tumor stage, grade, size, and lymph node metastasis. To study the relationship between UTRS level and the efficacy of different treatment modalities (such as surgery, chemotherapy, radiotherapy, immunotherapy, etc.). The completion of these studies will further deepen our understanding of this subgroup and further guide future clinical treatment.

Breast cancer is a highly heterogeneous disease, and conventional bulk sequencing or histopathological analysis can only provide average information about tumor tissue, and cannot reveal the characteristics of individual cells. By using single-cell technology, we can carry out multi-omics analysis of gene expression, genome, epigenetics and so on a single cell, so as to accurately identify different cell subtypes, and deeply understand the pathogenesis and progression of breast cancer (81, 82). Single-cell technology can simultaneously analyze tumor cells, immune cells, fibroblasts and other cell types, comprehensively characterize the cell composition and interaction of tumor microenvironment, and help reveal the complex relationship between tumor cells and microenvironment. Our research helps to discover biomarkers associated with drug targets, providing guidance for drug development and clinical trials.

However, it must be noted that this research does have certain limitations. First, there are some limitations to the TCGA and GEO databases we used. For example, data in TCGA and GEO come from different research institutions and experimental platforms, and there are differences in data collection and processing methods. Despite the large amount of data available from TCGA and GEO, sample sizes may still be insufficient for certain patient populations with rare cancer subtypes or specific clinical features. The collection of TCGA and GEO data is not a random sample, but is based on the needs and feasibility of the research project. This can lead to selection bias in the sample, i.e. patients included in the database may not be fully representative of the overall characteristics of all cancer patients. Cancers are highly heterogeneous, with significant differences in biological behavior, molecular characteristics, and prognosis among different subtypes of cancer. A prognostic model developed based on mixed cancer subtype data may not accurately predict the prognosis of patients with each subtype. In addition, the diversity of the sample is insufficient, which may not be able to comprehensively represent various types of BC patients, which may affect the universality of the study results. In addition, there are uneven tumor stages, and the distribution of samples at different stages may be uneven, which will interfere with the accurate analysis of the characteristics and rules of different tumor stages, and thus affect the reliability and generalization of the research results. In order to enhance the reliability and universality of the prognostic model, we are currently actively collecting independent clinical data sets, which will be derived from breast cancer patients in different regions and different hospitals, covering multiple subtypes and different clinical characteristics, to ensure the diversity and representativeness of the samples. And *in vitro* experiments were conducted. To ensure more reliable conclusions, these results should be confirmed by future research and cross-compared across different cancer types. For a more comprehensive validation, based on the potential therapeutic targets discovered in this study, such as C1 *UGDH*+TCs and CEBPD, we plan to conduct cell - line and animal - model experiments. After that, multiple functional assays will be carried out to explore their roles. We aim to clarify the functions and therapeutic effects of these targets, laying a solid foundation for pre - clinical and clinical trials.

## Data Availability

The data for this study come from the Gene Expression Omnibus (GEO) (https://www.ncbi.nlm.nih.gov/geo/) database. The GEO accession is GSE189357.

## Supplementary material

Supplementary Figure 1 The correlation between 15 modeled genes and survival rate was showed by the survival graph.

Supplementary Figure 2
**(A)** Volcano plots demonstrated the differentially expressed genes in the 5 cellular subpopulations. **(B)** The distribution of named genes of 5 subpopulations on UMAP was shown. **(C)** Heatmap demonstrated the Top 5 metabolism-related pathways with high correlation to the 5 cell subpopulations. **(D)** Heatmap demonstrated the correlation between the 5 cellular subpopulations of BC and their corresponding highly expressed genes. **(E)** Violin plot showed the expression of the 5 named genes in each of the 5 cellular subpopulations.

Supplementary Figure 3
**(A)** UMAP plot demonstrated the distribution of BC TCs over all TCs by fitting 2 differentiation trajectories through the proposed temporal order. **(B)** UMAP plot demonstrated Lineage 1-2 differentiation trajectories at the proposed temporal sequence. **(C)** Heatmap of GO-BP enrichment analysis demonstrated the correlation between the 5 cellular subpopulations of BC and their corresponding highly expressed pathways. **(D)** Scatterplot demonstrated the trajectories of the named genes of the 5 cellular subpopulations of BC TCs obtained after slingshot visualization of the changes over 2 Lineages.

Supplementary Figure 4
**(A)** Heatmap demonstrated afferent and efferent signaling intensities for the full cellular interactions. **(B)** Hierarchical plot showed the interactions between TCs and other cells in the CDH signaling pathway. Solid and hollow circles indicated source and target cell types, respectively. The edge color of the middle circle corresponded to the signaling source. **(C)** The centrality score of the CDH signal path network was shown through a heat map, showing the relative importance of each cell group. **(D)** Violin plot showed the expression of genes associated with the CDH signaling pathway in subpopulations of TCs and other cell types. **(E)** Heatmap showed cellular interactions in the CDH signaling pathway.

Supplementary Figure 5
**(A)** The survival graph showed the relationship between overall survival rate and UTRS. **(B)** The forest map showed a Multivariate analysis of the genes that made up the UTRS. HR>1 risk factor, HR<1 protective factor. **(C)** Nomogram predicted 1-year, 3-year, and 5-year overall survival based on age, high and low Risk score groups, and stages.

Supplementary Figure 6 InferCNV explored sc RNA-seq data from breast cancer cells to distinguish the cells that we want to study.

Supplementary Figure 7
**(A)** Left: The survival graph showed the relationship between overall survival rate and UTRS of GSE37751. Right: ROC curves showed the 1 -, 3 -, and 5-year OS in the GSE37751 dataset. **(B)** Left: The survival graph showed the relationship between overall survival rate and UTRS of GSE159956. Right: ROC curves showed the 1 -, 3 -, and 5-year OS in the GSE159956 dataset.

Supplementary Figure 8
**(A)** Dot plot showed the results of all GO-BP enrichment analyses. **(B)** GSEA scoring of GO-BP enrichment entries for differential genes showed the enrichment score values for different pathways. **(C)** Heatmap showed the correlation of the mutation profiles of the genes that made up the risk score group. **(D)** Mutation waterfall plot showed the differences in the top 20 most frequently mutated genes in somatic cells between the two groups. The upper bars indicated the mutation load for each sample, and the right bars indicated the total percentage of mutations in that gene in those samples. **(E)** Bar graph showed the results of predicting chromosome gains and losses in TCGA samples. Blue color indicated chromosome copy number gain; red color indicated chromosome copy number loss; and indicated no change in chromosome copy number. **(F)** Mutation waterfall plot showed mutations in the genes that made up the risk score group in the samples. The upper bars indicated the mutation load in each sample, and the right bars indicated the total percentage of mutations in that gene in those samples. **(G)** Overall description of the mutation profile of TCGA- BRCA patients. **(H)** Upper left: total mutations in all samples; Upper right: percentage of base substitutions and conversions, Ti referred to conversions (purines replaced by purines and pyrimidines replaced by pyrimidines) and Tv referred to subversions (substitutions between purines and pyrimidines); Lower: percentage bar graphs presented the mutations in each sample.

Supplementary Figure 9
**(A)** Dot plots of the expression of the 5 TF regulatory submodules of BC cell subpopulations. **(B, C)** Scatter plots demonstrated the fraction of variance across subpopulations and groups of 5 cell subpopulations of BC, respectively. **(D)** Ranking of regulators in 5 cell subpopulations of BC cells based on RSS.
