# Correction: Exposing the cellular situation: findings from single-cell RNA sequencing in breast cancer

**DOI:** 10.3389/fimmu.2025.1709624

**Published:** 2025-10-24

**Authors:** Gaofeng Ni, Xinhan Li, Wenyang Nie, Zhenzhen Zhao, Hua Li, Hongyan Zang

**Affiliations:** ^1^ Department of Breast Surgery, Yantaishan Hospital Affiliated to Binzhou Medical University, Yantai, China; ^2^ The First Clinical Medical College, Shandong University of Traditional Chinese Medicine, Jinan, Shandong, China; ^3^ Department of General Surgery, Affiliated Hospital of Youjiang Medical University for Nationalities, Baise, Guangxi, China; ^4^ Key Laboratory of Tumor Molecular Pathology of Baise, Affiliated Hospital of Youjiang Medical University for Nationalities, Baise, Guangxi, China

**Keywords:** single cell RNA sequencing, breast cancer, CEBPD, transcription factors, tumor microenvironment, metabolism

There was a mistake in **Figure 7F** as published. We mistakenly wrote CEBPD in **Figure 7F** as EGFR. The corrected **Figure 7F** appears below.

**Figure 7:**
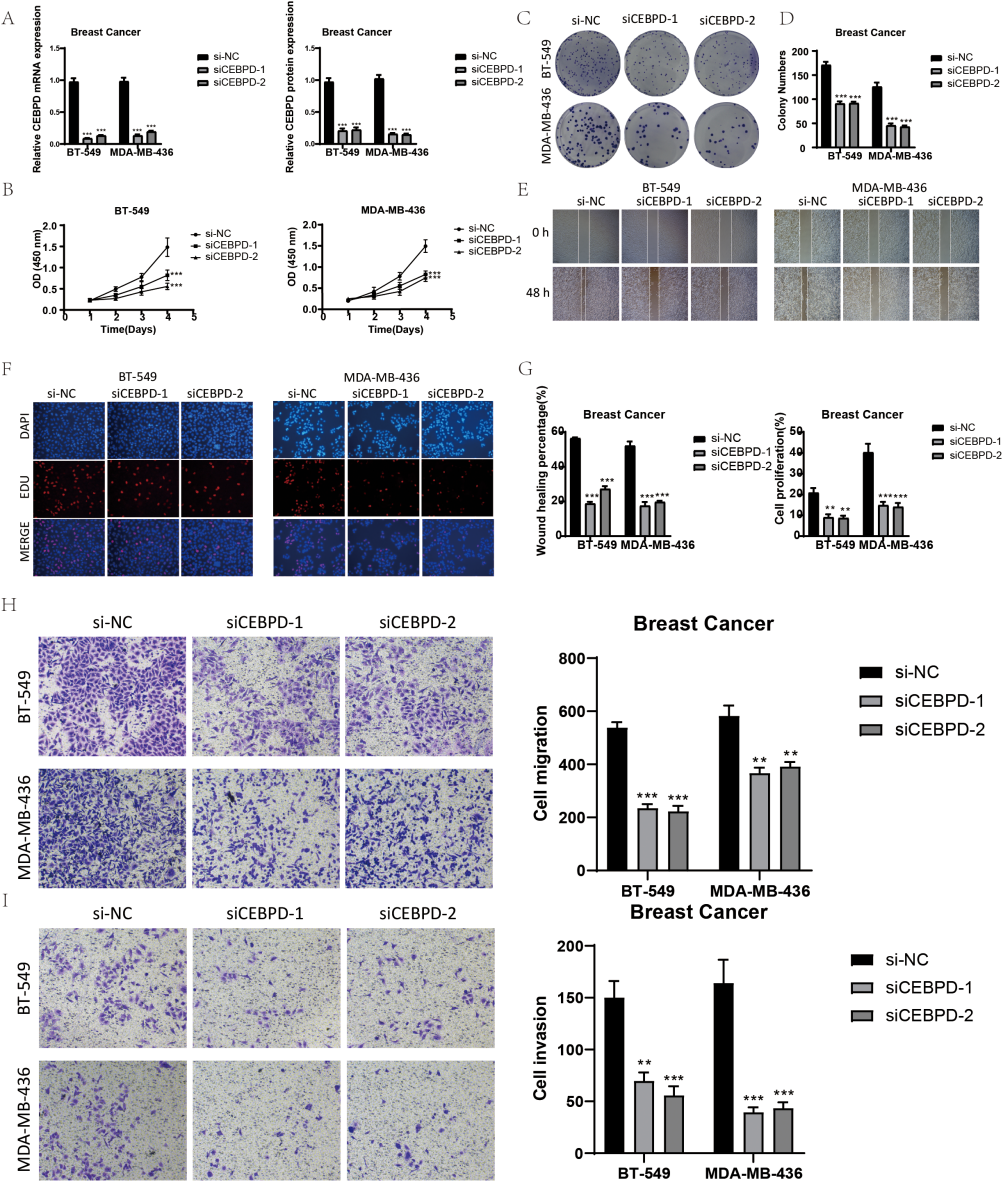
*In vitro* experimental validation of CEBPD. **(A)** After CEBPD knock-down, the expression levels of mRNA and protein decreased significantly. **(B)** CCK-8 detection showed that compared with the control group, the cell viability was significantly decreased after CEBPD knock-down. **(C, D)** The colony formation assay showed that the number of colonies decreased significantly after CEBPD was knocked down. **(E)** Scratch test showed that CEBPD knockdown inhibited cell migration. **(F)** EDU staining confirmed that CEBPD knock-down had inhibitory effect on cell proliferation. **(G)** Bar chart showed that the ability of cell migration and proliferation decreased significantly after CEBPD. **(H, I)** Transwell experiment showed that CEBPD knockdown inhibited the migration and invasion of TCs in BT-549 and MDA-MB-436 cell lines. *, p < 0.05; **, p < 0.0 1; ***, p < 0.001 indicates a significant difference, and NS indicates a non-significant difference.

The original version of this article has been updated.

