# Supplementary figures and images for: Exposing the cellular situation: findings from single cell RNA sequencing in breast cancer
Source: Front Immunol. 2025 Mar 6;16:1539074. doi: 10.3389/fimmu.2025.1539074 (PMC11922942; doi:10.3389/fimmu.2025.1539074)

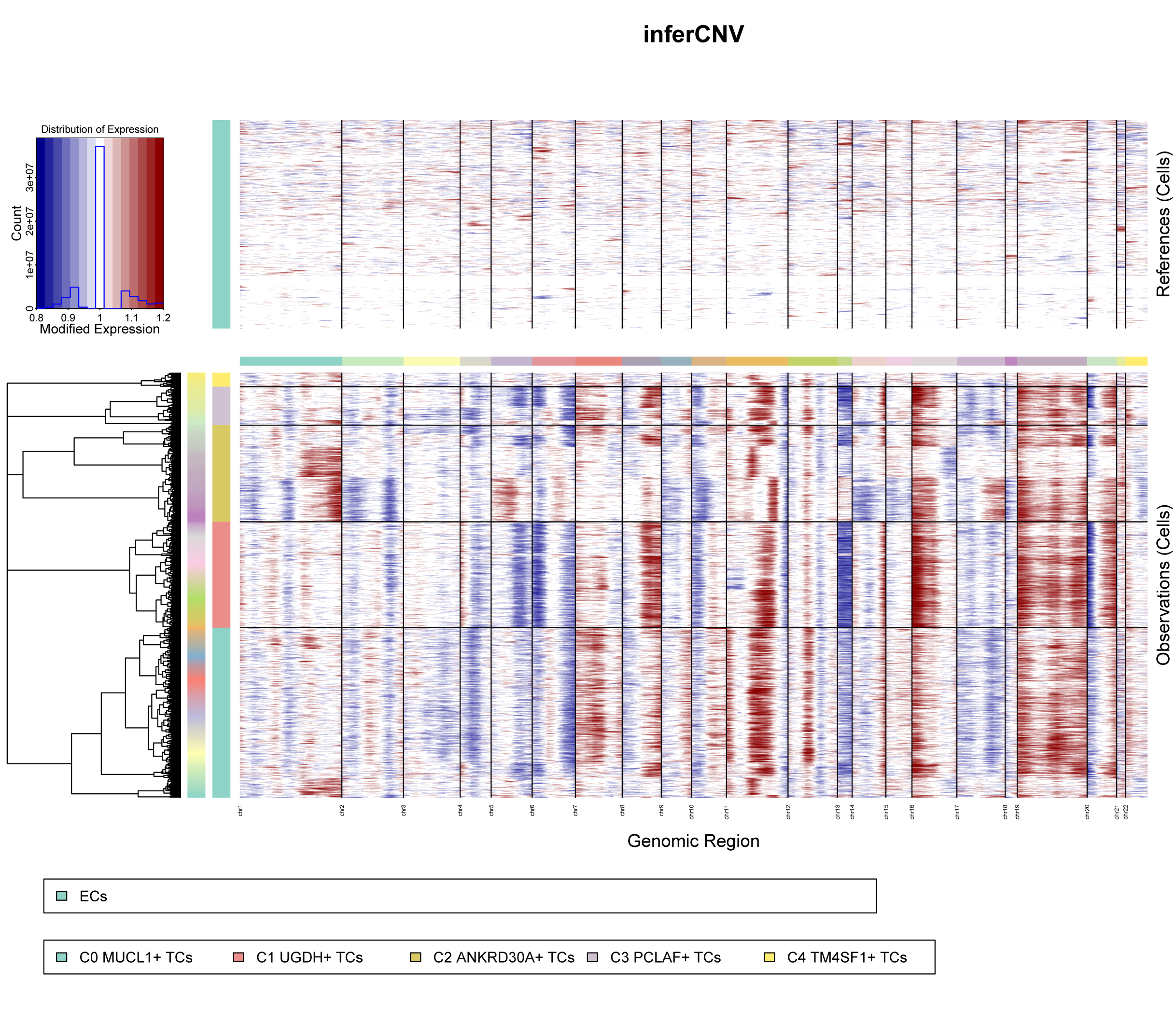

Supplement: Supplementary Figure 1 — The correlation between 15 modeled genes and survival rate was showed by the survival graph. [file Image1.tif]

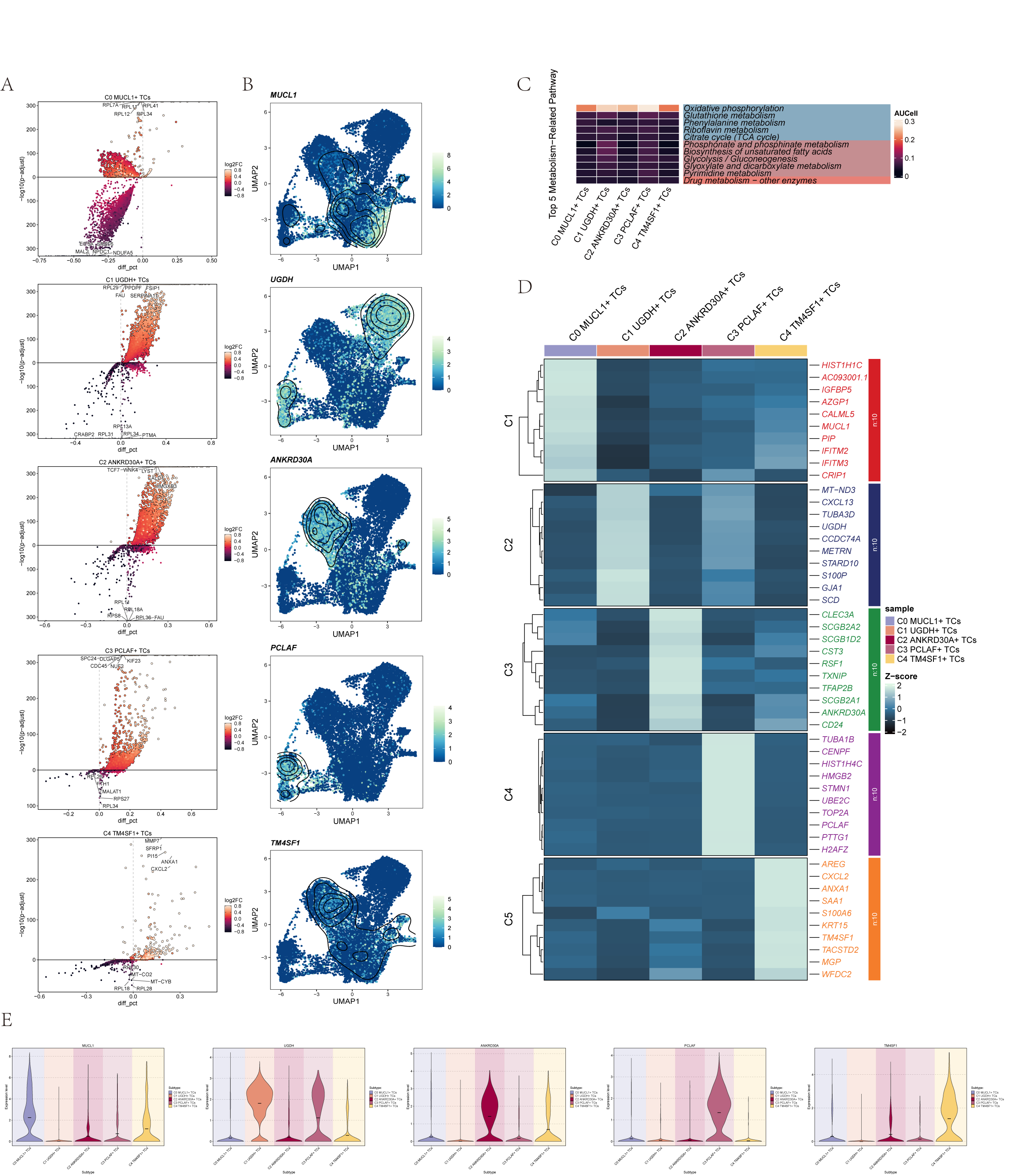

Supplement: Supplementary Figure 2 — (A) Volcano plots demonstrated the differentially expressed genes in the 5 cellular subpopulations. (B) The distribution of named genes of 5 subpopulations on UMAP was shown. (C) Heatmap demonstrated the Top 5 metabolism-related pathways with high correlation to the 5 cell subpopulations. (D) Heatmap demonstrated the correlation between the 5 cellular subpopulations of BC and their corresponding highly expressed genes. (E) Violin plot showed the expression of the 5 named genes in each of the 5 cellular subpopulations. [file Image2.tif]

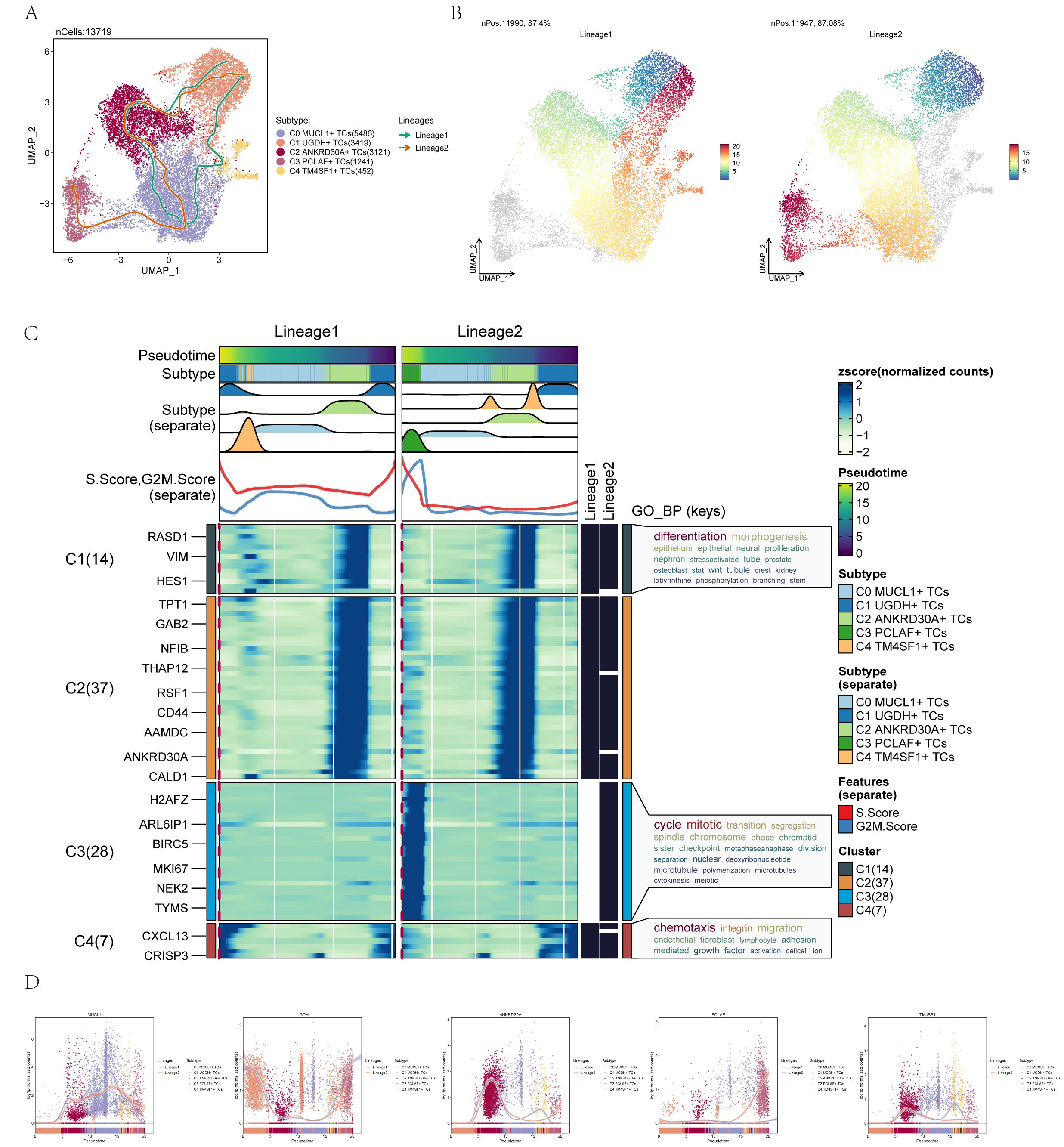

Supplement: Supplementary Figure 3 — (A) UMAP plot demonstrated the distribution of BC TCs over all TCs by fitting 2 differentiation trajectories through the proposed temporal order. (B) UMAP plot demonstrated Lineage 1-2 differentiation trajectories at the proposed temporal sequence. (C) Heatmap of GO-BP enrichment analysis demonstrated the correlation between the 5 cellular subpopulations of BC and their corresponding highly expressed pathways. (D) Scatterplot demonstrated the trajectories of the named genes of the 5 cellular subpopulations of BC TCs obtained after slingshot visualization of the changes over 2 Lineages. [file Image3.tif]

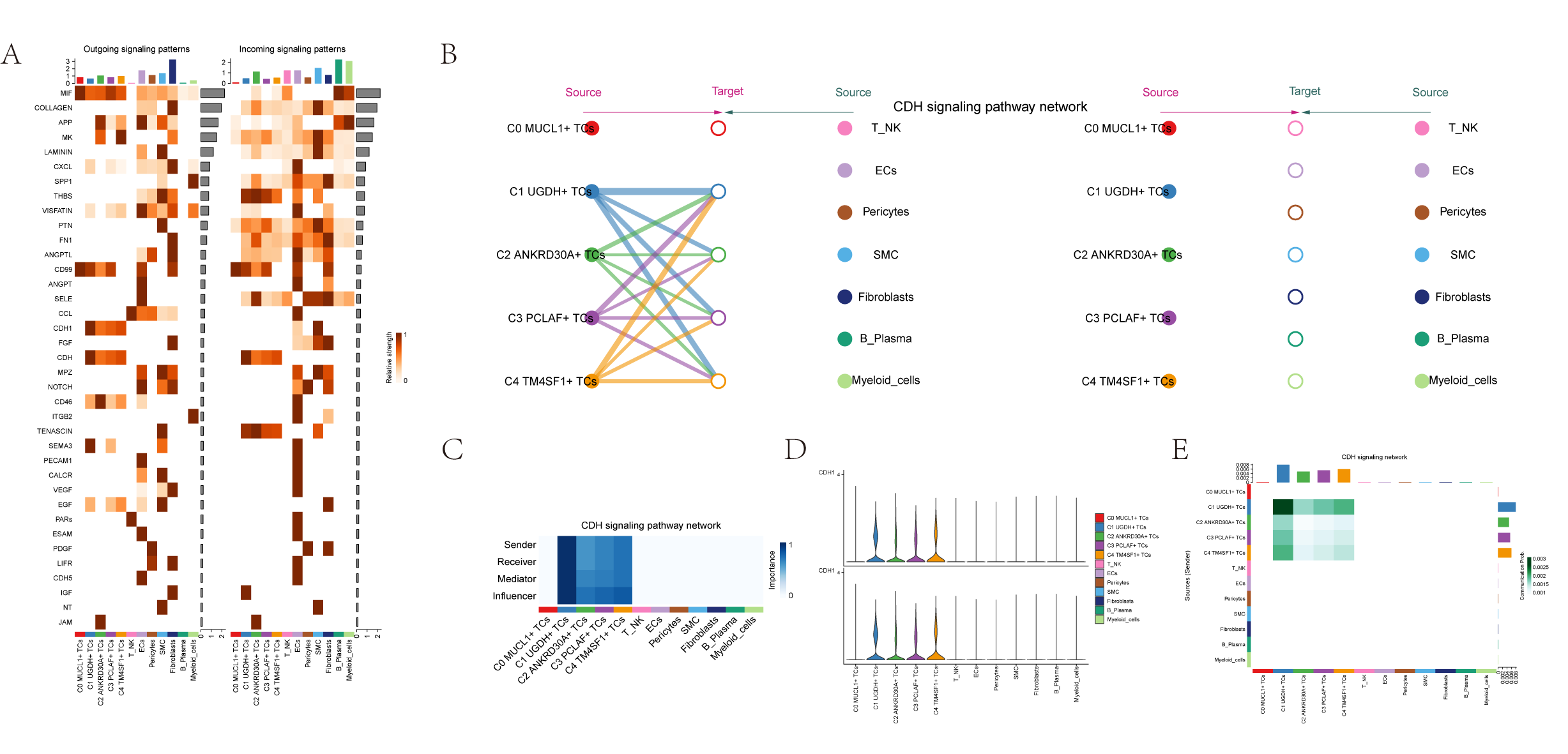

Supplement: Supplementary Figure 4 — (A) Heatmap demonstrated afferent and efferent signaling intensities for the full cellular interactions. (B) Hierarchical plot showed the interactions between TCs and other cells in the CDH signaling pathway. Solid and hollow circles indicated source and target cell types, respectively. The edge color of the middle circle corresponded to the signaling source. (C) The centrality score of the CDH signal path network was shown through a heat map, showing the relative importance of each cell group. (D) Violin plot showed the expression of genes associated with the CDH signaling pathway in subpopulations of TCs and other cell types. (E) Heatmap showed cellular interactions in the CDH signaling pathway. [file Image4.tif]

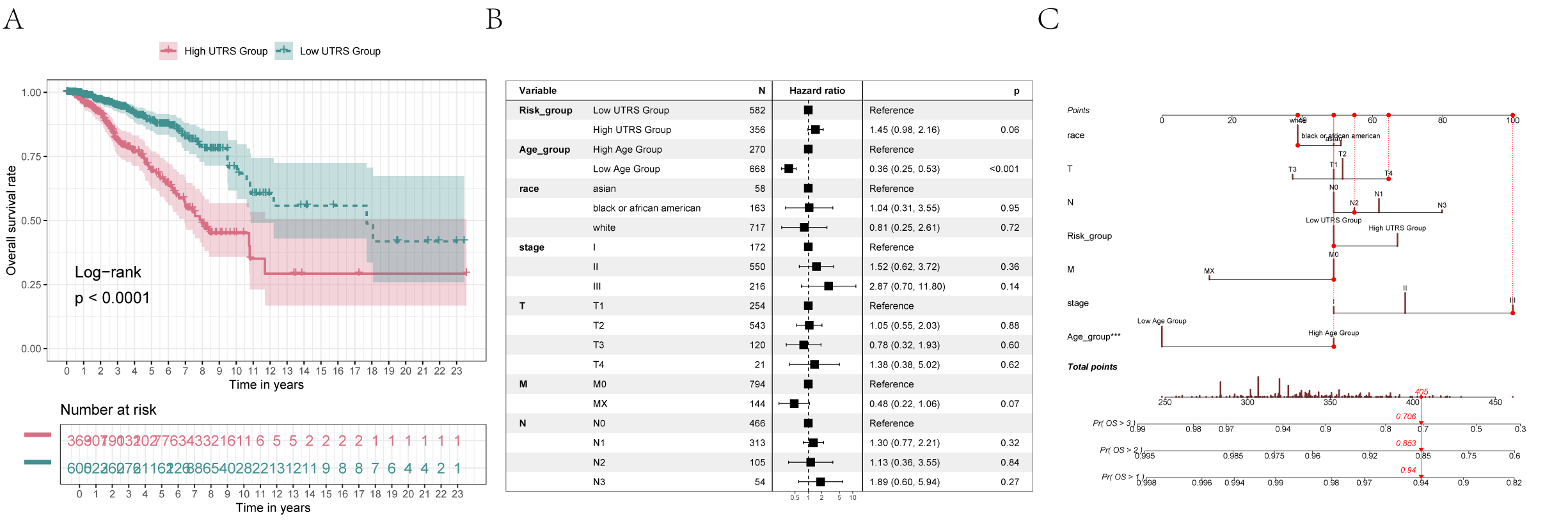

Supplement: Supplementary Figure 5 — (A) The survival graph showed the relationship between overall survival rate and UTRS. (B) The forest map showed a Multivariate analysis of the genes that made up the UTRS. HR>1 risk factor, HR<1 protective factor. (C) Nomogram predicted 1-year, 3-year, and 5-year overall survival based on age, high and low Risk score groups, and stages. [file Image5.tif]

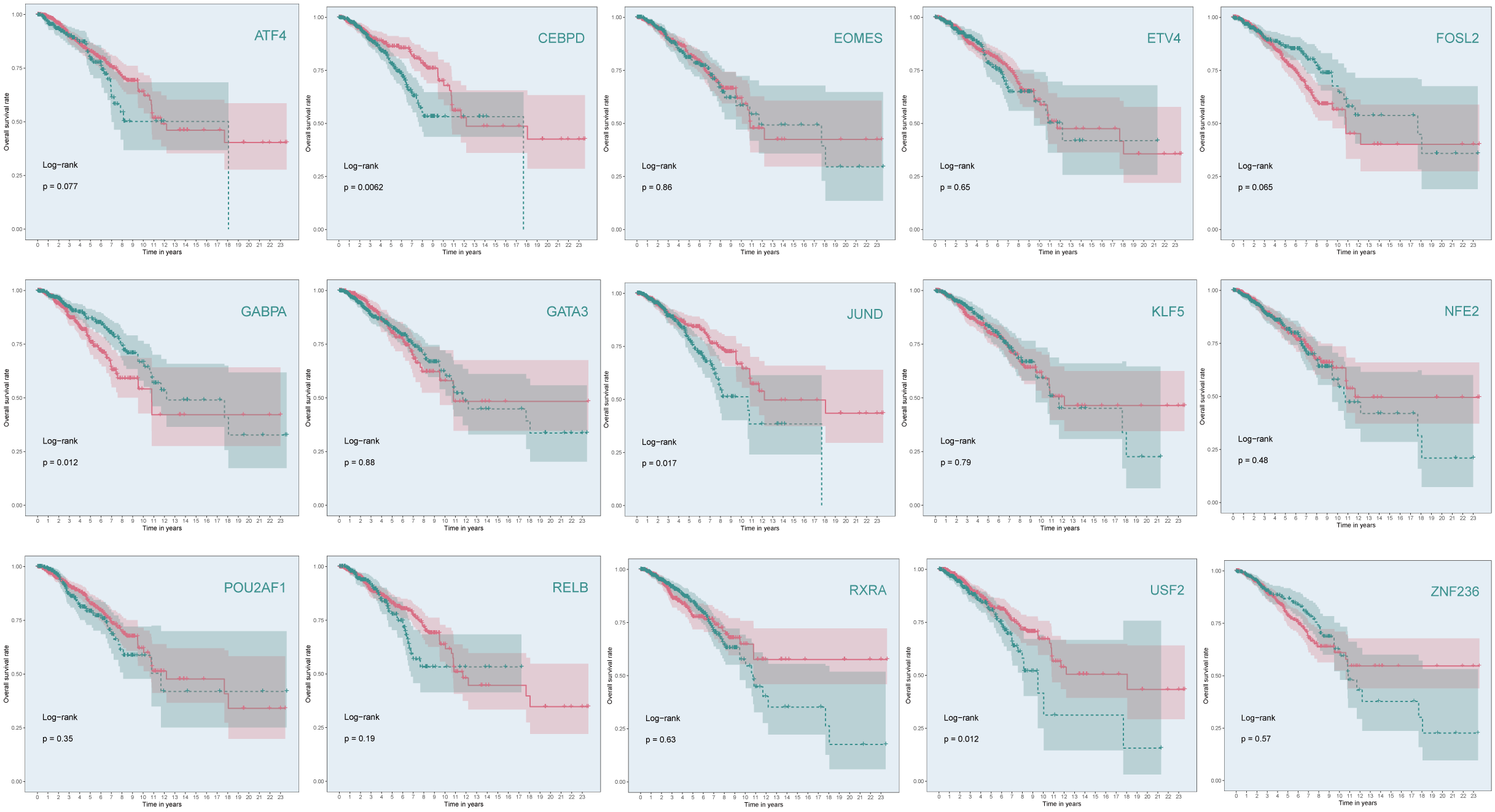

Supplement: Supplementary Figure 6 — InferCNV explored sc RNA-seq data from breast cancer cells to distinguish the cells that we want to study. [file Image6.tif]

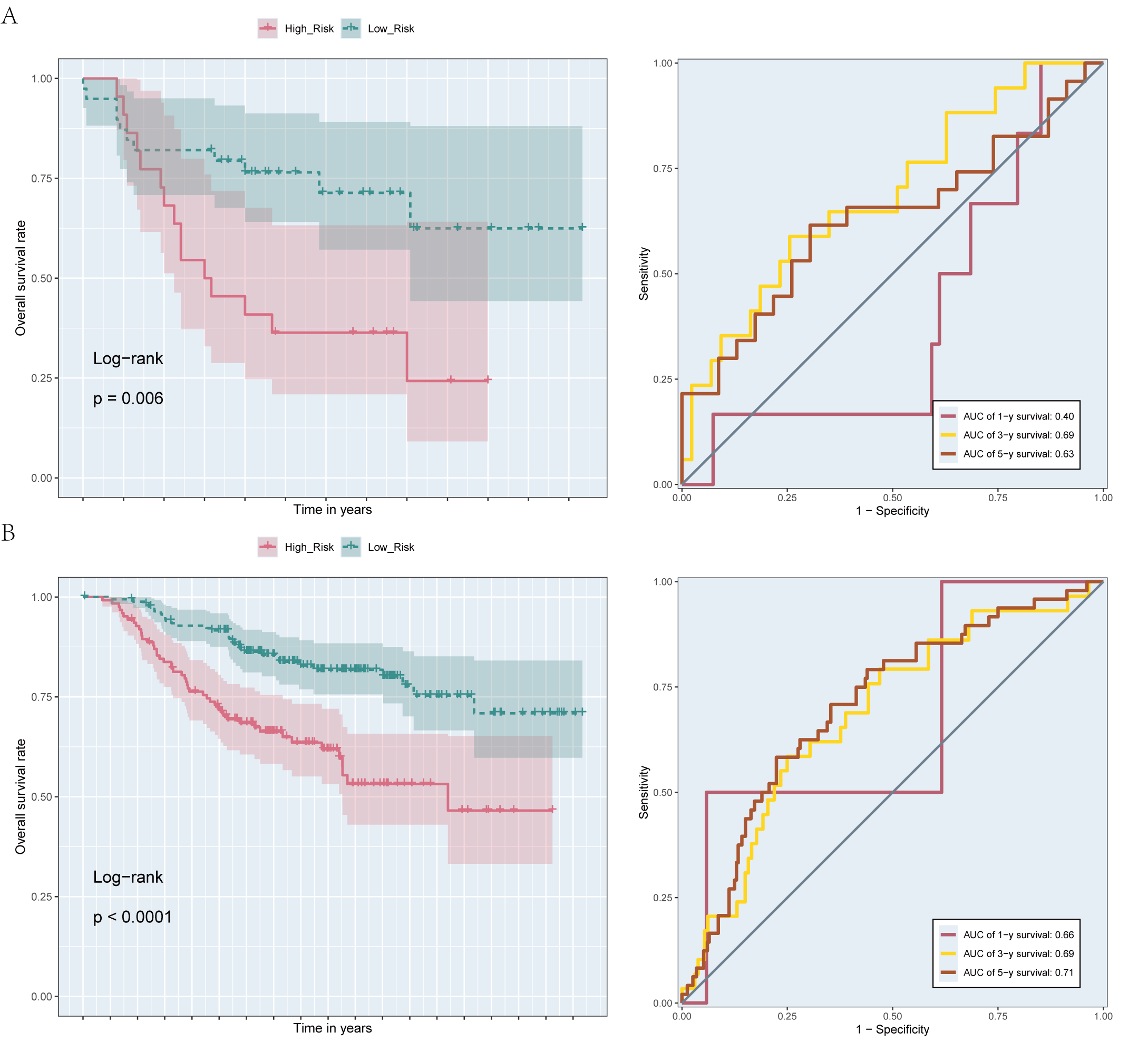

Supplement: Supplementary Figure 7 — (A) Left: The survival graph showed the relationship between overall survival rate and UTRS of GSE37751. Right: ROC curves showed the 1 -, 3 -, and 5-year OS in the GSE37751 dataset. (B) Left: The survival graph showed the relationship between overall survival rate and UTRS of GSE159956. Right: ROC curves showed the 1 -, 3 -, and 5-year OS in the GSE159956 dataset. [file Image7.tif]

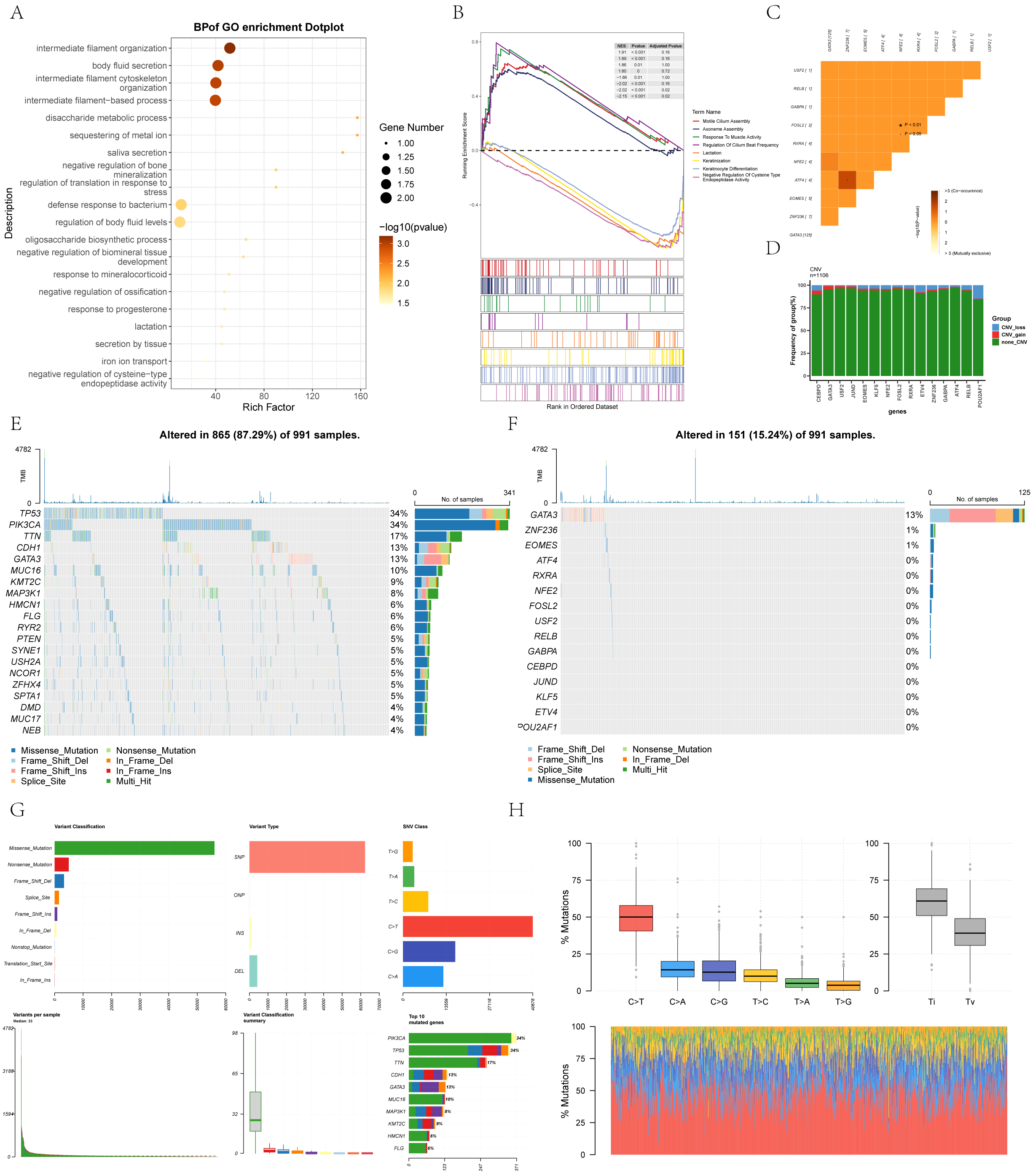

Supplement: Supplementary Figure 8 — (A) Dot plot showed the results of all GO-BP enrichment analyses. (B) GSEA scoring of GO-BP enrichment entries for differential genes showed the enrichment score values for different pathways. (C) Heatmap showed the correlation of the mutation profiles of the genes that made up the risk score group. (D) Mutation waterfall plot showed the differences in the top 20 most frequently mutated genes in somatic cells between the two groups. The upper bars indicated the mutation load for each sample, and the right bars indicated the total percentage of mutations in that gene in those samples. (E) Bar graph showed the results of predicting chromosome gains and losses in TCGA samples. Blue color indicated chromosome copy number gain; red color indicated chromosome copy number loss; and indicated no change in chromosome copy number. (F) Mutation waterfall plot showed mutations in the genes that made up the risk score group in the samples. The upper bars indicated the mutation load in each sample, and the right bars indicated the total percentage of mutations in that gene in those samples. (G) Overall description of the mutation profile of TCGA- BRCA patients. (H) Upper left: total mutations in all samples; Upper right: percentage of base substitutions and conversions, Ti referred to conversions (purines replaced by purines and pyrimidines replaced by pyrimidines) and Tv referred to subversions (substitutions between purines and pyrimidines); Lower: percentage bar graphs presented the mutations in each sample. [file Image8.tif]

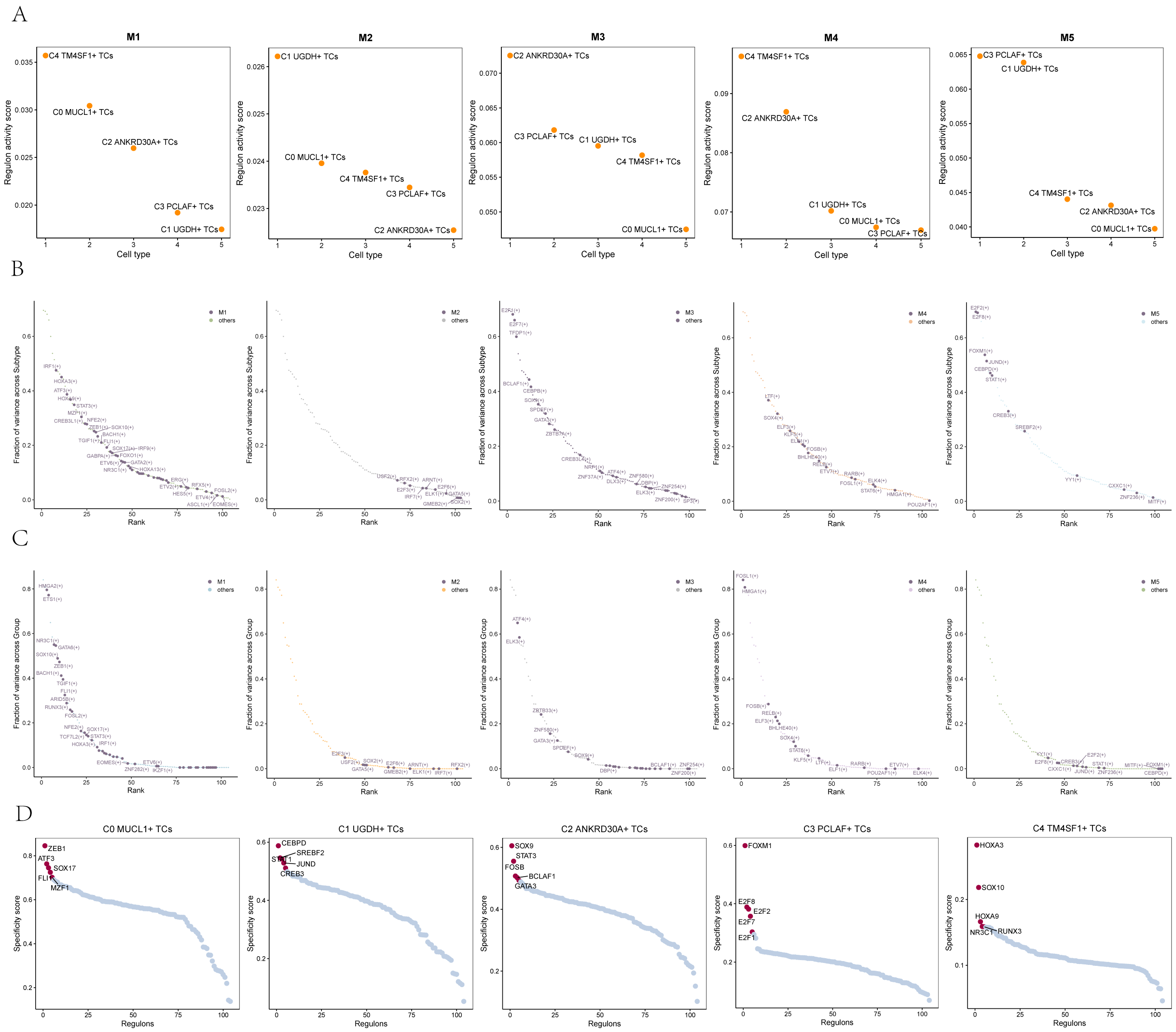

Supplement: Supplementary Figure 9 — (A) Dot plots of the expression of the 5 TF regulatory submodules of BC cell subpopulations. (B, C) Scatter plots demonstrated the fraction of variance across subpopulations and groups of 5 cell subpopulations of BC, respectively. (D) Ranking of regulators in 5 cell subpopulations of BC cells based on RSS. [file Image9.tif]
